# Assessing the Causal Role of Body Mass Index on Cardiovascular Health in Young Adults: Mendelian Randomization and Recall-by-Genotype Analyses

**DOI:** 10.1161/CIRCULATIONAHA.117.033278

**Published:** 2018-11-12

**Authors:** Kaitlin H. Wade, Scott T. Chiesa, Alun D. Hughes, Nish Chaturvedi, Marietta Charakida, Alicja Rapala, Vivek Muthurangu, Tauseef Khan, Nicholas Finer, Naveed Sattar, Laura D. Howe, Abigail Fraser, Debbie A. Lawlor, George Davey Smith, John E. Deanfield, Nicholas J. Timpson

**Affiliations:** 1 Medical Research Council Integrative Epidemiology Unit (K.H.W., L.D.H., A.F., D.A.L., G.D.S., N.J.T.); 2 Population Health Sciences, Bristol Medical School, Faculty of Health Sciences, University of Bristol, UK (K.H.W., L.D.H., D.A.L., G.D.S., N.J.T.).; 3 Vascular Physiology Unit, Institute of Cardiovascular Science, University College London, UK (S.T.C, M.C., A.R., V.M., T.K., N.F., J.E.D.).; 4 Cardiometabolic Phenotyping Group, Institute of Cardiovascular Science, University College London, UK (A.D.H., A.R., N.C.).; 5 Institute of Cardiovascular and Medical Sciences, British Heart Foundation (BHF) Glasgow Cardiovascular Research Centre, University of Glasgow, UK (N.S.).

**Keywords:** body mass index, cardiovascular system, causality, epidemiology, genetics

## Abstract

**Background:**

Body mass index (BMI) has been suggested to be causally related to cardiovascular health in mid-to-late life, but this has not been explored systematically at younger ages—nor with detailed cardiovascular phenotyping. Recall-by-Genotype (RbG) is an approach that enables the collection of precise phenotypic measures in smaller studies, while maintaining statistical power and ability for causal inference.

**Methods:**

In this study, we used a combination of conventional multivariable regression analysis, Mendelian randomization (MR), and subsample RbG methodologies to estimate the causal effect of BMI on gross-level and detailed cardiovascular health in healthy participants from the Avon Longitudinal Study of Parents and Children at age 17 years (N=1420–3108 for different outcomes) and an independent sample from the same cohort (for RbG) study at age 21 years (N=386–418).

**Results:**

In both MR and RbG analyses, results suggested that higher BMI causes higher blood pressure and left ventricular mass index in young adults (eg, difference in left ventricular mass index per 1 kg/m^2^ using MR: 1.07 g/m^2.7^; 95% CI, 0.62–1.52; *P*=3.87×10^-06^ and per 3.58 kg/m^2^ using RbG: 1.65 g/m^2.7^; 95% CI, 0.83–2.47; *P*=0.0001). Additionally, RbG results suggested a causal role of higher BMI on higher stroke volume (difference per 3.58 kg/m^2^: 1.49 mL/m^2.04^; 95% CI, 0.62–2.35; *P*=0.001) and cardiac output (difference per 3.58 kg/m^2^: 0.11 L·min^-1^·m^-1.83^; 95% CI, 0.03–0.19; *P*=0.01) but no strong evidence for a causal role on systemic vascular resistance or total arterial compliance. Neither analysis supported a causal role of higher BMI on heart rate.

**Conclusions:**

Complementary MR and RbG causal methodologies, together with a range of sensitivity analyses, suggest that higher BMI is likely to cause worse cardiovascular health, specifically higher blood pressure and left ventricular mass index, even in youth. Higher BMI also resulted in increased cardiac output in the RbG study, which appeared to be solely driven by stroke volume, as neither MR nor RbG analyses suggested a causal effect of BMI on heart rate. These consistent results support efforts to reduce BMI from a young age to prevent later adverse cardiovascular health and illustrate the potential for phenotypic resolution with maintained analytic power using RbG.

## Abstract 2

Supplemental Digital Content is available in the text.

**Clinical Perspective**

**What Is New?**

- The relationship between body mass index (BMI) and detailed cardiovascular measures has not been explored in young adults.
- We triangulated findings from 3 different analytic approaches with differing key sources of bias: conventional multivariable regression, Mendelian randomization, and a Recall-by-Genotype study design.
- The last is novel and exploits the random assortment of alleles through meiotic cell division at conception to inform genetically based recall and allows for the collection of extremely precise cardiovascular phenotypes that would otherwise be impractical at scale.
- This study illustrated the potential for phenotypic resolution with maintained analytic power and ability to draw causal inferences using Recall-by-Genotype.

**What Are the Clinical Implications?**

- Results suggested that higher BMI causes higher blood pressure (systolic and diastolic blood pressure, pulse pressure, and mean arterial pressure) and left ventricular mass index, the last suggesting adverse effects on cardiac structure, even in young adults.
- Recall-by-Genotype analyses also suggested that higher BMI increased cardiac output, which appeared to be solely driven by stroke volume, as neither Mendelian randomization nor Recall-by-Genotype analyses suggested a causal effect of BMI on heart rate.
- Our results support efforts to reduce BMI from a young age to prevent the development of precursors of long-term adverse cardiovascular health.

Higher body mass index (BMI) in adulthood is likely to cause numerous adverse cardiovascular risk factors and disease outcomes.^1–5^ These relationships may reflect long-term exposure to high adiposity and other comorbidities, which then result in adverse structural and functional cardiovascular changes that are different from adaptations encountered earlier in the disease’s evolution. Indeed, recent results from adult bariatric surgery patients provide supportive evidence for a causal role of greater adiposity on risk of major cardiovascular events.^3^ However, the nature of these relationships has been assessed predominantly in populations of adults, and no large studies have explicitly assessed the causal impact of BMI on detailed cardiovascular phenotypes in early life, where risk may emerge. Observational studies have reported associations between higher BMI and the presence of various subclinical markers of cardiovascular disease^6–8^; however, these can struggle to make a distinction between correlation and causation due to issues such as confounding or reverse causation.

One method for establishing evidence for causality in associations between an exposure and outcome of interest is Mendelian randomization (MR). This technique uses genetic variants as instrumental variables (or proxy measures) in otherwise observational epidemiological studies.^9^ The application of MR methodology has thus far provided evidence to support a causal role of higher BMI on increasing the risk of various cardiometabolic diseases, predominantly in large populations of adults.^2,4,5^ However, because of the technique’s requirement for large sample sizes to provide adequate statistical power, MR studies traditionally use routinely collected clinical measures or data generated from high-throughput technologies. To examine this potentially serious problem in the case of BMI and cardiovascular health of the young, detailed and precise subclinical measures of early structural and functional vascular adaptations are needed to help elucidate pathophysiology and disease etiology. However, these are not commonly carried out in large population studies, as they are expensive, are time-consuming, and require highly skilled operators. Therefore, such measures are limited in their availability for MR analyses, particularly within healthy young population samples.

Recall-by-Genotype (RbG) studies are an innovative extension of MR methodology, designed to improve study efficiency, enable genotype-driven deep-phenotyping, and improve causal inferences.^10^ This is achieved through the recall of participants based on already available genotypes that are known to be reliably correlated with exposures of interest (eg, BMI). Like MR and thus randomized, controlled trials, the random allocation of alleles at conception produces genotype groups that are theoretically independent of confounders and those that escape the problems of reverse causation. By recalling specific subgroups of a total sample with known exposure, the technique enables the efficient collection of precise phenotypic data that may be otherwise impractical at the scale necessary to achieve statistical power in MR analyses.^10^

Using data from ALSPAC (Avon Longitudinal Study of Parents and Children), we aimed to use both whole-sample MR and subsample RbG, alongside conventional multivariable regression analyses, to test the hypothesis that BMI causally influences variations in multiple clinically relevant measures of cardiovascular structure and function in adolescence and early adulthood.

## Methods

### Cohort Description

ALSPAC is a prospective birth cohort study investigating factors that influence normal childhood development and growth. The cohort and study design have been described in detail previously^11,12^ and are available at the ALSPAC website (http://www.alspac.bris.ac.uk). The study website contains details of all data that is available through a fully searchable data dictionary (http://www.bris.ac.uk/alspac/researchers/data-access/data-dictionary). Briefly, 14 541 pregnant women resident in a defined area of the South West of England, with an expected delivery date of April 1, 1991, to December 31, 1992, were enrolled to the cohort. Of these, 13 988 live-born children who were still alive 1 year later have been followed up to date with regular questionnaires and clinical measures, providing behavioral, lifestyle, and biological data. Ethical approval for the study was obtained from the ALSPAC Ethics and Law Committee and the Local Research Ethics Committee. Written informed consent was obtained from the parent/guardian and, after the age of 16 years, children provided written assent.

### Study Design

**Figure 1.**
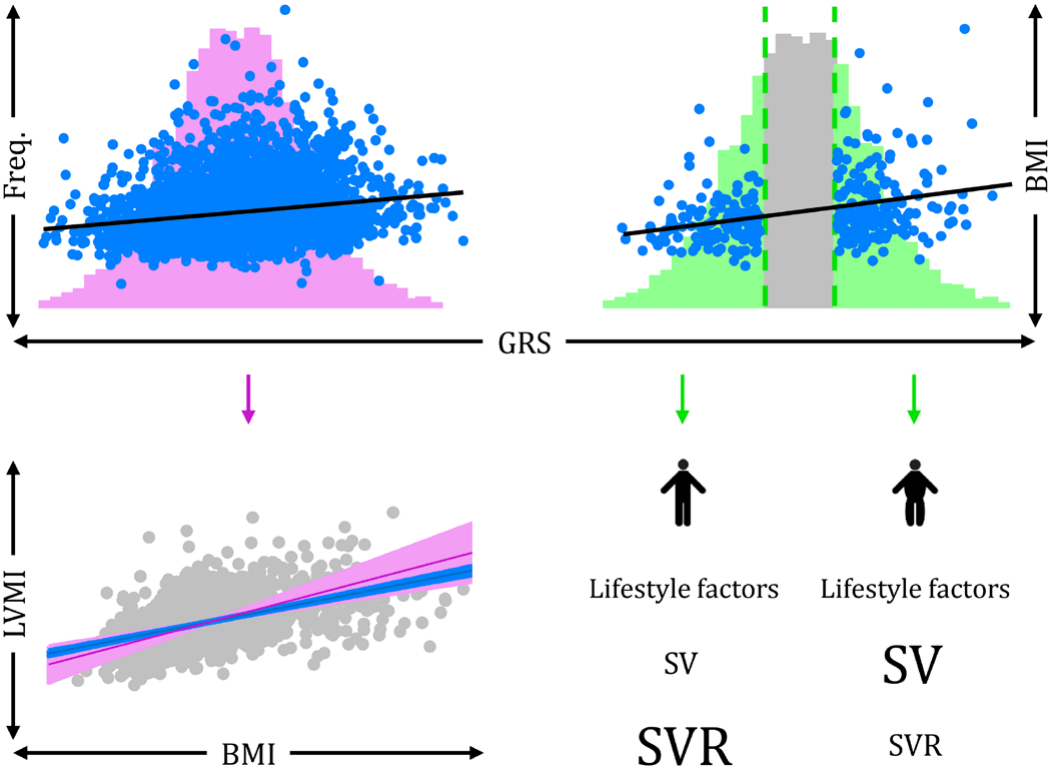
**Mendelian randomization and Recall-by-Genotype methodologies. Top left**, In a Mendelian randomization (MR) analysis, the entire distribution of a genetic risk score (GRS, in pink) is used as an instrumental variable (IV) for body mass index (BMI; observations in blue and association with GRS represented with black line) to assess the causal nature of association between BMI and cardiovascular phenotypes (eg, left ventricular mass index [LVMI]). **Bottom left**, Comparison of observational multivariable regression (blue) and MR-derived estimates (pink), showing a positive association between BMI and LVMI using both methodologies. **Top right**, Instead of using the entire distribution of a GRS, the Recall-by-Genotype (RbG) method creates genetically recalled samples from the tails of a GRS distribution (green), which are associated with BMI (observations in blue and association with GRS represented with black line). **Bottom right**, The RbG groups importantly show a difference in mean BMI between groups; however, there are no differences in confounding factors (equally sized “lifestyle factors” between groups). The RbG method therefore allows us to assess the change in detailed cardiovascular measures, obtained through precise techniques, between the recalled groups. Here, stroke volume (SV) is greater in those in the upper versus lower tail and systemic vascular resistance (SVR) is greater in the lower versus upper tail, both phenotypes of which were obtained through magnetic resonance imaging (MRI), which would not otherwise be feasible in large enough studies needed for MR methodology.

MR and RbG were used to test the hypothesis that BMI causally influences variations in multiple clinically relevant measures of cardiovascular structure and function in adolescence and early adulthood (Figure 1). First, we used a genetic risk score (GRS) comprising 97 BMI-associated single nucleotide polymorphisms (SNPs) shown to be robustly associated with BMI in the most recent genome-wide association study (GWAS),^13^ constructed using external weighting. This GRS was used as an instrumental variable (IV) within an MR framework to investigate the causal effect of BMI on a range of vascular measures collected from those who attended the 17-year clinic and underwent echocardiography (as part of the GRACE study).^13^ Estimates were compared with results obtained through observational analysis of the same associations. Second, we used data collected in a RbG framework to reproduce these findings and further explore their underlying mechanisms through the extensive phenotyping of a smaller group of independent individuals, recalled specifically on a genome-wide GRS distribution, constructed from results from the largest available GWAS of BMI conducted by Speliotes et al^14^ (at the initiation of recruitment to the RbG study) to explain the maximum possible proportion of variance in BMI (see Methods in the online-only Data Supplement).

Of those with full genetic data and consent (N=8350), individuals were invited to the RbG study based on the lower and upper ≈30% of the genome-wide GRS distribution.^14^ Of those invited (N=2071), 419 individuals were successfully recalled at an average age of 21 years (see Methods and Figures I–III in the online-only Data Supplement).

**Figure 2.**
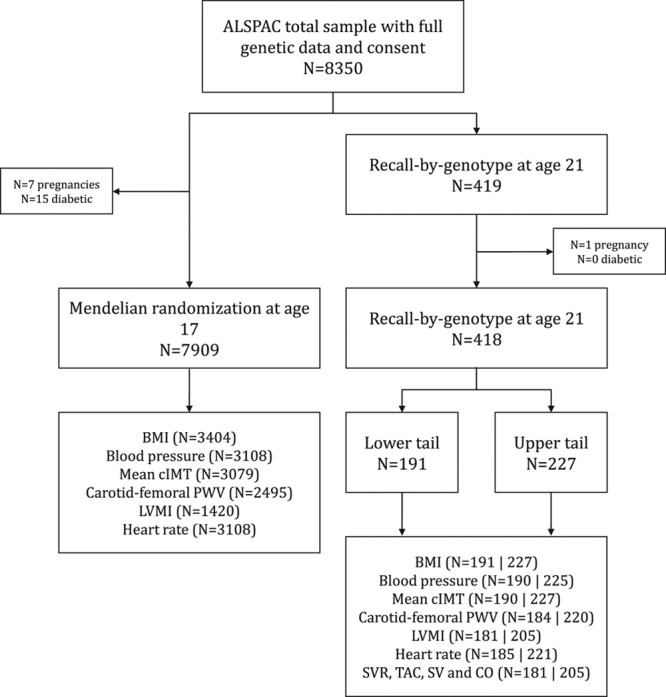
**Flow of samples used for Mendelian randomization and Recall-by-Genotype studies.** The total number of individuals in ALSPAC with full genetic data and consent was 8350. Of these, 418 individuals were used in the Recall-by-Genotype (RbG) study, based on the lower and upper ≈30% of a continuous genome-wide genetic risk score (GRS) distribution for body mass index (BMI), constructed on the basis of results from a genome-wide association study (GWAS) of BMI.^14^ A total of 191 were within the lower tail and 227 were in the upper tail. The independent sample of 7909 individuals was used in MR analyses at age 17 years, which used a GRS comprising 97 SNPs (and constructed using external weighting) shown to be associated with BMI from a large-scale GWAS.^13^ The number of individuals with available data on the exposure (BMI) and on both BMI and cardiovascular outcomes are also presented (with numbers in the lower and upper tail of the RbG sample separated by “|”). ALSPAC indicates the Avon Longitudinal Study of Parents and Children; BMI, body mass index; cIMT, carotid intima-media thickness; CO, cardiac output; LVMI, left ventricular mass index; PWV, pulse wave velocity; SV, stroke volume; SVR, systemic vascular resistance; and TAC, total arterial compliance.

We excluded data of all females who were pregnant or individuals who had diabetes mellitus at both the 17-year clinic (N=7 pregnancies and 15 diabetics) and the 21-year recall (N=1 pregnancy and 0 diabetics). After these exclusions, 418 individuals were used in the 21-year RbG analyses and, of these, all had measured BMI, and the sample sizes of those with cardiovascular measures ranged between 386 to 418 (Figure 2). In the independent sample of 7909 individuals that was used for MR analyses at age 17 years, 3404 had data on BMI and, of these, the sample sizes of those with cardiovascular measures ranged between 1420 to 3108 (Figure 2).

### Measures of Adiposity at Ages 17 and 21 Years

At both ages, height was measured to the nearest centimeter using a stadiometer (SECA 213, Birmingham, UK) and weight to the nearest 0.1 kg, unshod and in light clothing, using electronic weighing scales (Marsden M-110, Rotherham, UK). BMI was calculated as weight (kg) divided by height squared (m^2^).

### Cardiovascular Phenotypes at Age 17 Years

The following cardiovascular phenotypes were used in MR analyses to assess the causal role of BMI on cardiovascular health at age 17 years: blood pressure and heart rate, carotid-femoral pulse wave velocity (PWV), carotid intima-media thickness (cIMT), and left ventricular mass index (LVMI). In addition, detailed phenotypes were measured using magnetic resonance imaging (MRI) at age 21 years (RbG study), including LVMI, stroke volume (SV), cardiac output (CO), systemic vascular resistance, and total arterial compliance. See Methods in the online-only Data Supplement for details on phenotypic measurement.

### Confounders

Because of potentially confounding effects, the following variables were added as covariates in observational analyses: maternal education and household occupation, plus current smoking status and the most recent records of physical activity and, where available, dietary intake. See Methods in the online-only Data Supplement for details on measures of these covariates.

### Statistical Analyses

#### Preanalysis Transformations and Adjustments

As the distribution of residuals from the linear regression of BMI on carotid-femoral PWV was positively skewed, values of these variables were log-transformed for analyses. For interpretation, differences and CIs were back-transformed in all tables and figures and presented as the mean percentage difference in carotid-femoral PWV. Left ventricular mass measured at each age was indexed to height to the power of 2.7 (LVMI).^15^ To assess the impact of adiposity on central vascular measures over and above that caused by stature,^16^ systemic vascular resistance and total arterial compliance were adjusted for height using systemic vascular resistance×height^1.83^, total arterial compliance/height^1.83^, and both CO and SV measures were indexed to height by dividing CO by height^1.83^ and SV by height^2.04^.^17^ To assess the impact of missingness in our data, we compared the distributions of variables in those who had complete data (ie, no missing data in any variable) versus those included in our primary analyses. The magnitude of difference in all variables was negligible (Table I in the online-only Data Supplement); therefore, we present results based in the sample of individuals who had data on all necessary variables for each analysis. A priori, we planned to draw conclusions based on effect estimates and their CIs, rather than statistical tests using an arbitrary *P* value cut-off.^18,19^ For example, given 2 effects with the same point estimate—one with narrow CIs, the other with wider CIs that may even include the null—we describe both as showing the same effect but note that one is more imprecisely estimated and should be treated with more caution until replicated in a larger sample. We did not take account of multiple testing given the high correlation between our outcome variables. The use of “positive” and “inverse” throughout the text refer to directional association rather than clinical implication. Stata 14 (Stata Corp) and R (https://cran.r-project.org/) were used for all analyses.

#### Multivariable Regression

Observational associations between BMI and each cardiovascular phenotype at age 17 years were assessed using multivariable linear regression in 3 models: (1) unadjusted, (2) adjusted for age, sex, smoking status and dietary intake of the participant, household social class, and maternal education, and (3) additionally adjusted for physical activity (added as a separate model due to sample size). Associations of the confounders with BMI, each cardiovascular measure, and the weighted GRS at age 17 years were tested using linear regression.

#### Mendelian Randomization

The externally weighted GRS used as an IV for BMI in MR analyses was generated from the 97 SNPs shown to be reliably associated with BMI in the most recent GWAS conducted by the Genetic Investigation of ANthropometric Traits (GIANT) consortium.^13^ To generate the GRS, the dosage of each BMI-increasing allele at each locus in ALSPAC was weighted by the external effect size of the variant in the GWAS results.^20^ The doses were then added together and multiplied by the average external effect size of all the SNPs on BMI to reflect the number of average BMI-increasing alleles carried by each individual.

Two-stage least squares analysis was performed using the GRS as an IV for BMI at age 17 years to estimate β-coefficients and standard errors from MR methodology (*ivreg2* command in Stata). *F* statistics for the first-stage regression between the GRS and BMI were examined to check the instrument validity, satisfying the assumption that the GRS was sufficiently associated with the exposure.^21^ The Durbin-Wu-Hausman test for endogeneity was used to compare multivariable regression and IV effect estimates (*ivendog* command in Stata).^22^

#### Recall-by-Genotype

Linear regression was used to assess the association of the RbG group allocation (upper versus lower ≈30% of the genome-wide GRS distribution, as described above) with BMI and each of the cardiovascular phenotypes measured at age 21 years. Each estimate therefore represents the mean difference in each variable with the corresponding mean difference in BMI between RbG groups (equivalent to a *t* test). The associations of the confounders with BMI, each cardiovascular measure at age 21 years, and the RbG group allocation were tested using linear and logistic regression, where appropriate.

### Sensitivity Analyses

Both blood presssure (BP) and heart rate are correlated with other cardiovascular measures, namely carotid-femoral PWV, cIMT, and LVMI.^23–25^ To assess the causal association between BMI and these cardiovascular measures, accounting for BP and heart rate, we took the residuals of the regression between each of these variables and both systolic BP (SBP) and heart rate, and repeated the MR and RbG main analyses using these residuals.

#### MR Analyses

Additionally, evidence suggests that some of the cardiovascular phenotypes used in these analyses are not independent of height.^15,26,27^ To account for this and the inconsistent residual correlation between BMI and height throughout the lifecourse,^28^ we assessed the association between the weighted GRS on height at age 17 years and explored the impact of adjustment for height and height-squared on the association between the weighted GRS and BMI. We also adjusted both multivariable regression and MR analyses for height and height-squared and compared these to the main analyses.

The use of multiple alleles in MR analyses increases the potential for unbalanced pleiotropic effects (where the inclusion of invalid genetic instruments has an aggregate effect in one particular direction).^9,20,29^ Where pleiotropy is perfectly balanced, an informative GRS is sufficient in an MR analysis, but this method is less able to cope with unbalanced pleiotropic effects. To investigate the validity of the weighted GRS as an IV, the MR-Egger approach was used to detect and accommodate violations of the MR assumptions.^29^ The intercept of the MR-Egger test can be interpreted as an estimate of the average pleiotropic effect across the genetic variants, with a nonzero intercept term indicating overall unbalanced or directional pleiotropy. MR-Egger estimates were compared to those obtained from the inverse-variance weighted and weighted median methods,^29,30^ which provide estimates of the causal effect of BMI on cardiovascular phenotypes under varying assumptions of instrument validity. As in the main analyses, the estimates of the association between each SNP and BMI were obtained from an independent external source, as to not induce weak instrument bias in a 2-sample MR setting.^31^

Previous studies have suggested that the 97 SNPs used to construct the weighted GRS may have different properties in non-European populations.^32^ Therefore, we performed a sensitivity analysis using a weighted GRS that was restricted to the genetic variants that were associated with BMI in the analysis of only people of European descent and excluded those that only reached genome-wide significance in 1 sex or stratum (n=77) in the GIANT consortium.^13^ Additionally, a previous study in a large sample based in the UK suggested exclusion of 3 variants owing to pleiotropy (rs11030104, rs13107325, and rs3888190) and 3 SNPs that are not in Hardy-Weinberg equilibrium (*P*<1×10^−6^; rs17001654, rs2075650, and rs9925964).^32^ Therefore, as a sensitivity analysis, we excluded these additional SNPs, resulting in an IV consisting of 71 SNPs.

Additionally, to assess the validity of the genome-wide GRS (used to recruit individuals to the RbG study, based on the Speliotes et al^14^ GWAS), MR analyses were conducted using the same genome-wide GRS as an IV for BMI, scaled to represent the same difference in mean BMI per unit increase as compared with the Locke et al^13^ GRS, comprising 97 SNPs, used in main MR analyses.

## Results

The MR cohort were 17.8 years old (SD = 0.4), consisted of 47.8% females, and had an average BMI of 22.7 kg/m^2^ (SD = 4; Table 1). In the RbG study, individuals were 21.5 years old (SD = 0.9) and had an average BMI of 24.5 kg/m^2^ (SD = 5.7), and 65.8% were females (Table 2).

**Table 1.**
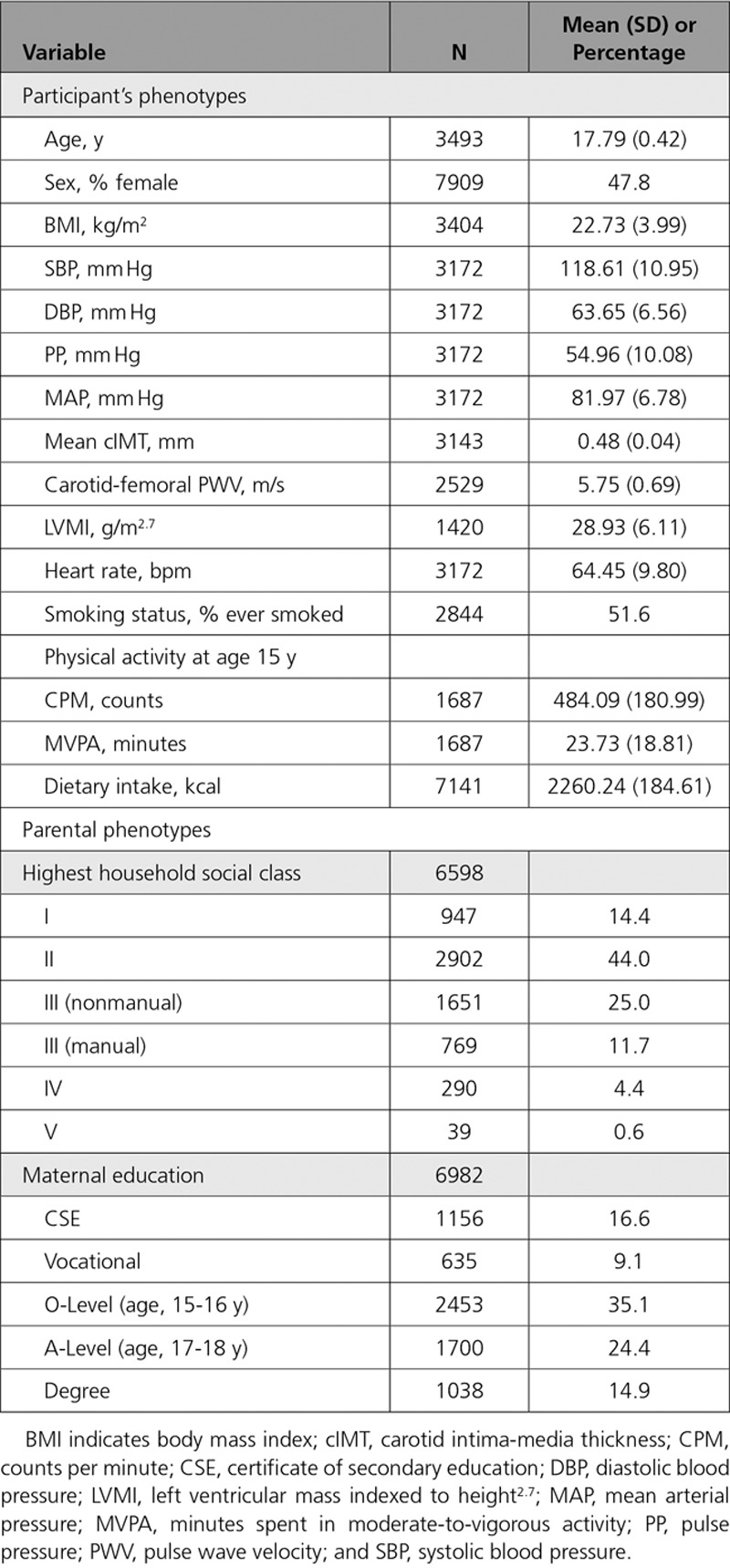
Descriptive Statistics for ALSPAC 17-Year Clinic

**Table 2.**
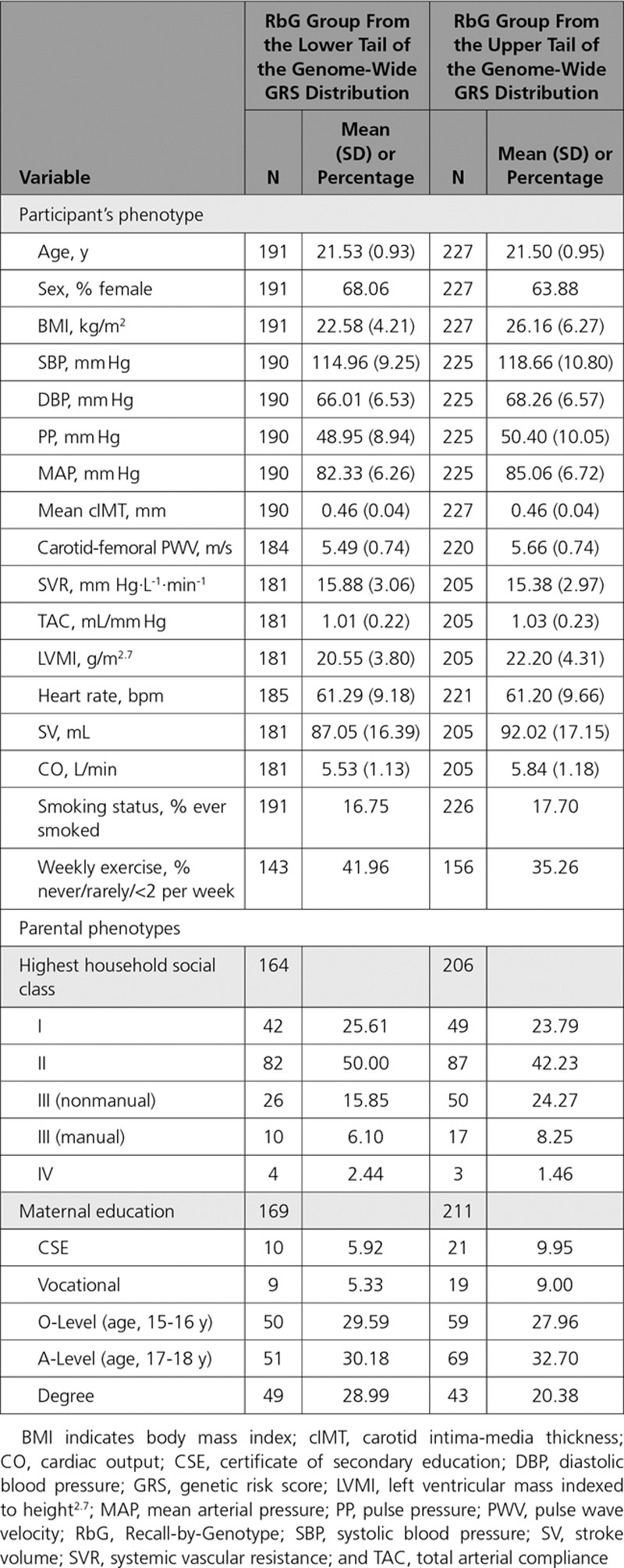
Descriptive Statistics for ALSPAC 21-Year RbG Group

### Multivariable Regression

Multivariable regression analyses provided evidence for positive associations of measured BMI with SBP, diastolic BP (DBP), pulse pressure, mean arterial pressure (MAP), LVMI, and heart rate at age 17 years, as well as an inverse association with carotid-femoral PWV (Table 3).

**Table 3.**
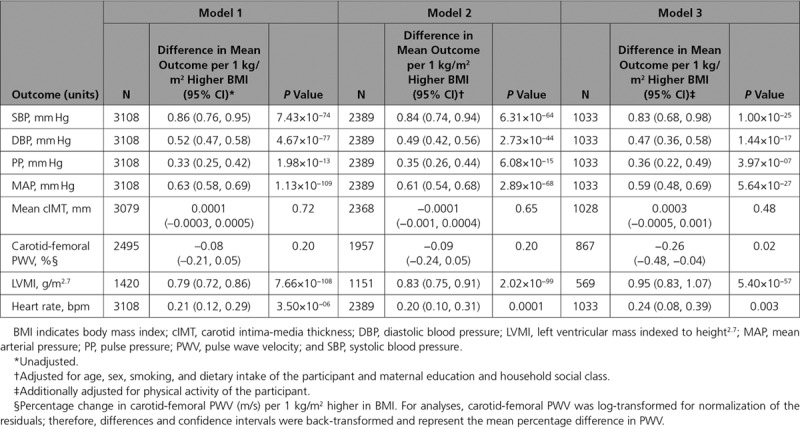
Multivariable Regression Associations Between BMI and Cardiovascular Phenotypes in ALSPAC 17-Year Clinic

### Confounder Analyses

BMI and all the cardiovascular phenotypes were associated with most of the confounding factors including highest household social class, maternal education, age, sex, smoking status, and dietary intake (Table II–X in the online-only Data Supplement). Unlike the direct measure of BMI and the cardiovascular phenotypes, the GRS was not associated with a majority of confounders (Table XI in the online-only Data Supplement). However, there was evidence for an association between the GRS and dietary intake.

### Mendelian Randomization

Each allele increase in the weighted GRS (comprising 97 SNPs) was associated with a 0.12 kg/m^2^ (95% CI, 0.10–0.14; *P*=9.53×10^-28^) higher BMI, explaining 3% of the variance (Figure I in the online-only Data Supplement). There was evidence for a positive effect of each kg/m^2^ higher BMI on SBP (difference: 0.79 mm Hg; 95% CI, 0.30–1.28; *P*=0.002), DBP (difference: 0.29 mm Hg; 95% CI, 0.0002–0.59; *P*=0.05), pulse pressure (difference: 0.49 mm Hg; 95% CI, 0.03–0.96; *P*=0.04), MAP (difference: 0.46 mm Hg; 95% CI, 0.16–0.75; *P*=0.002), and LVMI (difference: 1.07 g/m^2.7^; 95% CI, 0.62–1.52; *P*=3.87×10^−06^; Table 4). *F* statistics for these analyses ranged from 36 to 123, suggesting reasonable instrument strength. There was no strong evidence that the results from MR analyses were different from those from the multivariable regression analyses (all *P* values for comparison >0.12).

**Table 4.**
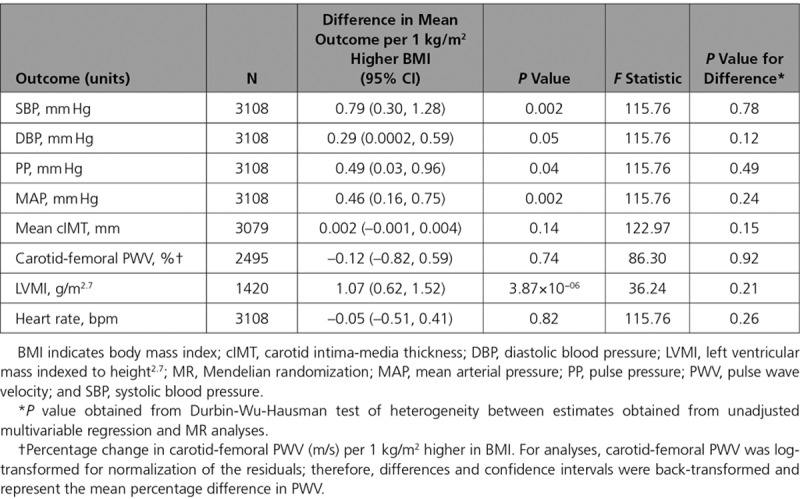
Mendelian Randomization Analyses of the Association Between BMI and Cardiovascular Phenotypes in ALSPAC 17-Year Clinic

### Recall-by-Genotype on BMI

Difference in mean BMI between RbG groups was 3.58 kg/m^2^ (95% CI, 2.53–4.63; *P*=6.09×10^−11^; Table 5, Figure II in the online-only Data Supplement).

**Table 5.**
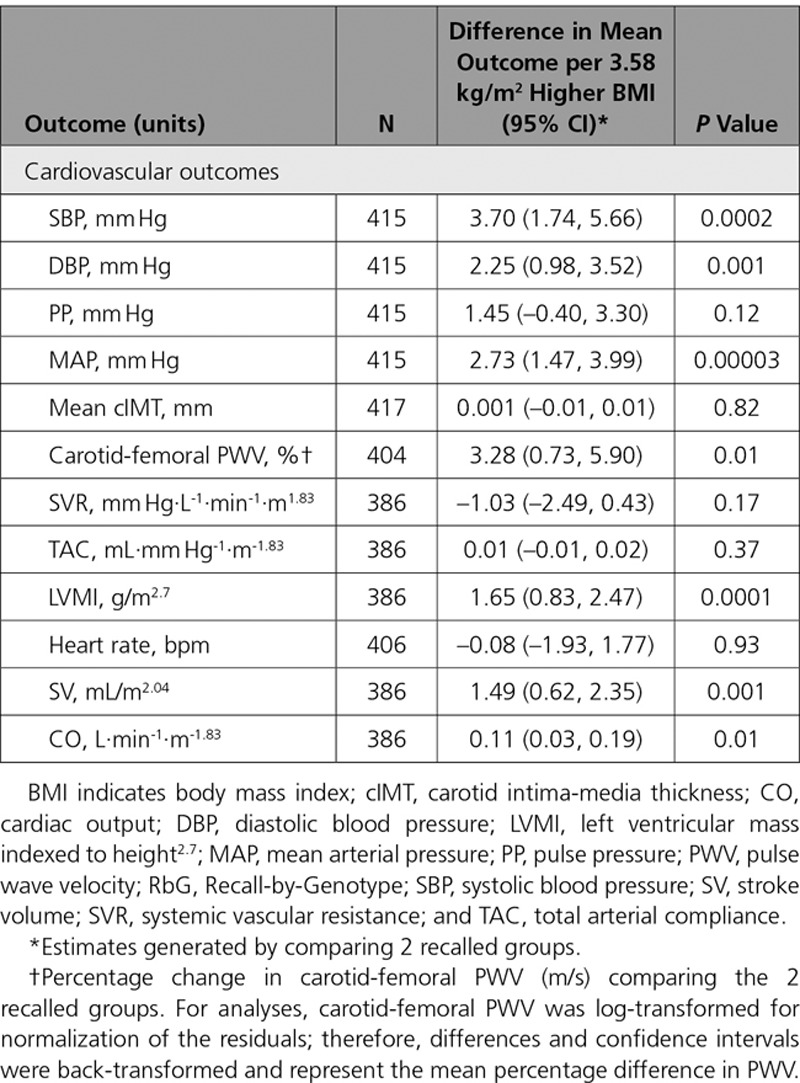
Association Between RbG Groups and Cardiovascular Measures in ALSPAC 21-Year RbG Group

### Confounder Analyses

Measures of both BMI and cardiovascular outcomes at age 21 years were associated with a majority of confounders, including highest household social class, maternal education, age, sex, smoking status, physical activity, and dietary intake (Tables XII-XXIV in the online-only Data Supplement). There was no strong evidence that the RbG group allocation was associated with confounders (Table XXV in the online-only Data Supplement).

### Recall-by-Genotype and Cardiovascular Phenotypes

Of the cardiovascular measures that overlapped between the 2 methods (MR and RbG), the RbG groups were associated with higher SBP (difference in mean between upper versus lower RbG groups: 3.70 mm Hg; 95% CI, 1.74–5.66; *P*=0.0002), DBP (difference: 2.25 mm Hg; 95% CI, 0.98–3.52; *P*=0.001), MAP (difference: 2.73 mm Hg; 95% CI, 1.47–3.99; *P*=0.00003), and carotid-femoral PWV (difference: 3.28%; 95% CI, 0.73–5.90%; *P*=0.01; Table 5).

**Figure 3.**
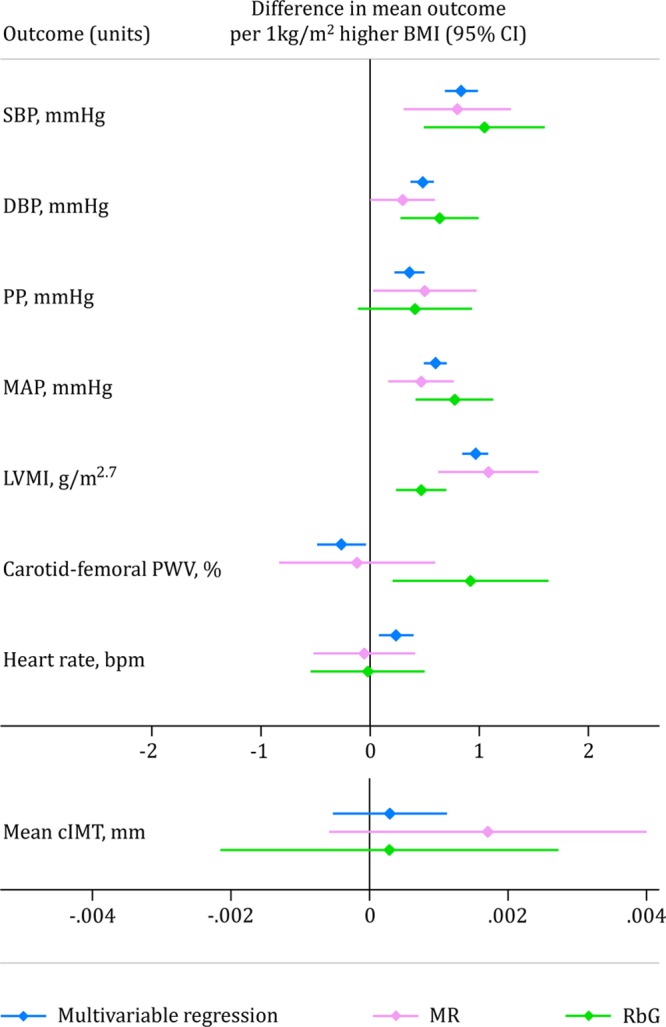
**Comparison of estimates of all overlapping cardiovascular phenotypes available at both ages.** Estimates represent the difference in mean outcome per 1 kg/m^2^ higher body mass index (BMI; graphs are separated by scale similarities) from multivariable regression (blue), Mendelian randomization (MR; pink), and Recall-by-Genotype (RbG; green) methodologies. cIMT indicates carotid intima-media thickness; DBP, diastolic blood pressure; LVMI, left ventricular mass index; MAP, mean arterial pressure; PP, pulse pressure; PWV, pulse wave velocity; and SBP, systolic blood pressure.

Scaling the effect estimates to represent each kg/m^2^ higher BMI, as in the MR analyses, these results are equivalent to a 1.03-mm Hg higher SBP, 0.63-mm Hg higher DBP, 0.76-mm Hg higher MAP, and 0.91% higher carotid-femoral PWV (Figure 3). There was therefore consistency between effect estimates on the overlapping phenotypes at both ages (ie, each 1 kg/m^2^ higher BMI had a causal effect of similar magnitude on SBP, DBP, and MAP), while showing no association with heart rate or cIMT (Figure 3). However, there was evidence for a positive causal effect of BMI on carotid-femoral PWV in RbG analyses at age 21 years that was not evident in MR analyses at 17 years.

In addition to these cardiovascular measures, the RbG framework allowed the collection of more precise cardiovascular phenotypes. Of those specifically collected in the RbG arm of this work, there was evidence for a causal role of higher BMI on MRI-derived LVMI (difference in mean between upper versus lower RbG groups: 1.65 g/m^2.7^; 95% CI, 0.83–2.47; *P*=0.0001), SV (difference: 1.49 mL/m^2.04^; 95% CI, 0.62–2.35; *P*=0.001), and CO (difference: 0.11 L·min^-1^·m^-1.83^; 95% CI, 0.03–0.20; *P*=0.01), with no strong evidence of a difference in systemic vascular resistance or total arterial compliance (Table 5).

### Sensitivity Analyses

After adjusting for SBP and heart rate, both MR and RbG results for the effect of BMI on cIMT, carotid-femoral PWV, and LVMI were mostly consistent with the main analysis (Table XXVI in the online-only Data Supplement). The 1 exception was the positive effect of BMI on carotid-femoral PWV shown in RbG analysis, which attenuated to the null after adjustment for SBP and heart rate (difference: 1.56%; 95% CI, −0.71 to 3.89%; *P*=0.18).

#### MR Analyses

The weighted GRS (comprising 97 SNPs) used in MR analyses was not associated with height or height-squared at age 17 years. Adjusting for both height and height-squared made no difference to the association between the GRS and BMI at age 17 years (Table XXVII in the online-only Data Supplement), multivariable regression analyses (Table XXVIII in the online-only Data Supplement), or MR analyses (Table XXIX in the online-only Data Supplement).

The MR-Egger test provided no strong evidence for unbalanced pleiotropic effects for any genetic variant included within the GRS on any cardiovascular outcome (all *P* values for the intercept ≥0.24; Table XXX in the online-only Data Supplement). The MR effect estimates from inverse-variance weighted, MR-Egger, and weighted median analyses for the causal effect of BMI on the cardiovascular phenotypes were largely consistent with the main analyses, though the effect estimate of BMI on SBP did not agree for the weighted median analyses in comparison with the main MR, inverse-variance weighted, and MR-Egger estimates, albeit with very wide CIs (Table XXX in the online-only Data Supplement and Figures III–VII in the online-only Data Supplement).

Both the IV containing 77 SNPs (Figure VIII in the online-only Data Supplement) and 71 SNPs (Figure IX in the online-only Data Supplement) were associated with BMI to a comparable extent as the GRS, comprising the full set of 97 SNPs based on the Locke et al^13^ GWAS (Table XXXI in the online-only Data Supplement) and produced similar results to the main analyses (Tables XXXII and XXXIII in the online-only Data Supplement, respectively). Similarly, when the genome-wide GRS (initially used to recall individuals to the RbG study and based on the Speliotes et al GWAS^14^) was implemented in MR analyses, the GRS was associated with a comparable change in BMI (Table XXXI in the online-only Data Supplement, Figure X in the online-only Data Supplement) and produced similar results to main MR analyses (Table XXXIV in the online-only Data Supplement).

## Discussion

In a large cohort of young adults, we used 2 complementary analyses (MR and RbG) to investigate the causal effect of higher BMI on measures of cardiovascular structure and function and compared these to adjusted multivariable regression results. Alongside MR analyses, RbG exploits the random assortment of alleles through meiotic cell division at conception to inform genetically based recall and further allows for the collection of extremely precise cardiovascular phenotypes that would otherwise be impractical at a scale required to infer causality using MR analyses. Results suggest that higher BMI causes higher BP (SBP, DBP, PP, and MAP) and LVMI, the last suggesting adverse cardiac structure, even in young adults.

Regarding the cardiovascular phenotypes that were used across MR, RbG, and multivariable regression analyses in the current study, our results are consistent with previous observational studies in children and adults.^33–35^ For example, a recent study using MR methodology provided evidence that higher BMI and central adiposity (measured by BMI-adjusted waist-hip ratio) increased the risk of coronary heart disease, type 2 diabetes mellitus, and echocardiographic measures indicative of left ventricular hypertrophy (such as the log Cornell product^36^). This highlights not only the consistency of our current findings but also how our analyses build on the current literature with respect to the phenotypes measured and methods used.^35^ The similarity of findings across these methods, given different sources of bias between the MR and RbG on the one hand^29^ and multivariable regression on the other,^9^ strongly supports causality in this instance. If further sustained through adulthood, these effects of higher BMI are likely to increase cardiovascular disease risk and cardiovascular disease–specific mortality in later life.^37–40^

Previous multivariable regression results from smaller observational studies in children and adolescents have found higher BMI to be associated with faster carotid-femoral PWV and thicker cIMT.^41–44^ In contrast to this, our 3 methods gave results that did not support a causal effect for cIMT. This suggests that previous studies may have been influenced by residual confounding or bias, for which we have been better able to control here. This conclusion is also supported to some extent by analysis of the same variables at a younger age in an observational context.^33^ With less consistent results across our approaches, higher BMI was associated with slower carotid-femoral PWV at age 17 years (ie, healthier PWV) in multivariable regression analysis and a faster carotid-femoral PWV (ie, worse PWV) in RbG analysis, with MR analysis showing a null association (Figure 3). Indeed, a previous study suggested that the relationship between BMI and PWV may be inverse in youth and becomes positive at older ages.^45^ This discrepancy could indeed reflect a time-varying role of BMI on PWV but could also be a result of differential carotid-femoral PWV measurement techniques used between the 2 ages (a Vicorder device used at age 17 years versus the SphygmoCor Vx device at age 21 years) or chance.

As might be expected, higher BMI resulted in increased CO in our RbG study and, although contrary to other observational studies at this age,^46,47^ this appeared to be solely driven by SV, as neither our MR or RbG analyses suggested a causal effect of BMI on heart rate. It is possible that previously reported associations between BMI and heart rate may be a result of unmeasured confounding. The BMI-mediated change in SV (and consequently CO) seen at age 21 years is therefore likely to at least partially account for the cardiac hypertrophy and higher BP that we see in the data analyzed here.

A key strength of this work is the comparison of results from confounder-adjusted multivariable regression and MR, but also the ability to use the RbG framework to extend this analysis and use detailed cardiovascular phenotyping to explore causal associations. This is the first use of RbG for BP and cardiac structure, where we could compare results directly to multivariable regression and MR within the same general population. The consistency for shared phenotypes between results found from RbG (with 386–418 participants) to those from MR (with 1420–3108 participants) suggests that this approach is valid and statistically efficient. Furthermore, the RbG method allowed the collection of precise cardiovascular phenotypes that would otherwise not have been possible in sample sizes required for MR, while allowing causal inference. For example, we were able to explore the impact of BMI on MRI-derived SV and CO, measures that are prohibitively expensive to undertake and thus often inaccurately estimated in several thousands of participants. As opposed to other methods, the RbG study design used here afforded the possibility of collecting more precise MRI-derived cardiovascular phenotypes, which are more likely to accurately capture clinically relevant variation in cardiovascular health.

In contrast to these strengths, it is of course the case that both the MR and RbG analyses may be biased if IV analyses assumptions are violated.^9^ These require, first, that the genetic instruments need to be robustly related to the exposure (here, BMI). We used variants in both MR and RbG that have been shown to be genome-wide significant and replicated; the first-stage *F*-statistics, a measure of instrument strength, were high for all the MR analyses. Second, it is assumed that confounders of the observational BMI-cardiovascular outcome association are not related to the genetic instrument. There is empirical evidence that this is unlikely to be the case and, for observed confounders, we demonstrated this in our analyses here.^48^ Third, it is assumed that there is no independent path from the genetic instrument to the outcomes other than through BMI, which may result from horizontal pleiotropy. Although we aimed to make best use of all available data, our main analyses included the use of an aggregate GRS comprising all 97 SNPs associated with BMI, which may increase the possibility of horizontal pleiotropy. Therefore, we used the best available methods for MR analyses with multiple IVs to test and, to some extent, account for pleiotropy. Across this range of sensitivity analyses (including the MR-Egger and weighted median approaches, as well as limiting the GRS to different subsets of genetic instruments), results were broadly similar to main analyses, lending more confidence to the causal estimates and direction of effect with higher BMI, suggesting that these were not largely driven by horizontal pleiotropy.

Although the RbG approach enabled sampling from the lower and upper ≈30% of a genome-wide GRS, which produced a difference of ≈3.5 kg/m^2^ in BMI, the genome-wide nature of the score could be considered less refined than the GRS used in MR analyses, comprising 97 SNPs shown to be robustly associated with BMI in a large meta-analyses of GWASs.^13^ Despite this, sensitivity analyses performed showed that the genome-wide GRS provided comparable results with MR analyses. Of those who participated in the RbG study, 386 individuals had full data on MRI-derived cardiovascular phenotypes. Although these measures were not present in the full sample (N=418), this is one of the largest collections of MRI-derived phenotypes in individuals of this age that were collected in a manner that allowed causal inference of the effect of BMI on these phenotypes. Second, we used overlapping phenotypes (such as blood pressure, pulse rate, and carotid-femoral PWV) in both the MR and RbG analyses primarily for comparison of causal estimates between the 2 ages. Thus, the current study provides both comparative and novel cardiovascular phenotypes in the context of adiposity.

One complication in some of the sensitivity analyses performed (specifically, adjusting for variables including BP, heart rate, and height in multivariable and MR analyses) is the potential for inducing collider or selection bias.^49,50^ However, because of the overall consistency in effect estimates generated from the various sensitivity analyses, this is unlikely to be the case. In addition, it is possible that some of the differences in effect size of BMI on cardiovascular outcomes (for example, carotid-femoral PWV) between ages may relate to either the difference in age at which the analyses were undertaken or phenotyping methods used at these ages. Given the small range of some of the cardiovascular outcomes (for example, cIMT) in these young individuals and the potentially small effect size of BMI, power to detect such small effect sizes in this context may be limited. Further, we adjusted for a range of potentially confounding factors in multivariable regression analyses, but even in such a comprehensive longitudinal cohort it can be difficult to accurately measure or observe (and therefore appropriately account for) all confounders. Indeed, this illustrates the need for better methods (such as MR and RbG used here) to assess the causal nature of the association between BMI and cardiovascular health, which aim to overcome such limitations. Finally, in all analyses, we only included participants with complete data on all variables used in the specific model (ie, complete data on BMI, outcome and all confounders in multivariable analyses and on genetic instruments, BMI and outcome in Mendelian randomization). This assumes that missing data are missing at random. The similarity in characteristics between those with complete data and those with any missing data suggested that this assumption is unlikely to be violated.

With this innovative study design, using complementary multivariable regression, MR, and RbG analyses, together with a range of sensitivity analyses, results suggest that higher BMI is likely to cause adverse levels of BP and LVMI, implying adverse cardiac structure, even in youth. RbG analyses also suggest that higher BMI results in increased CO, which appeared to be solely driven by SV, as neither MR nor RbG analyses suggested a causal effect of BMI on heart rate. These findings support efforts to reduce BMI from a young age, with the aim of attenuating the development of precursors of long-term adverse cardiovascular outcomes. Such efforts may help prevent the development of additional cardiac and peripheral vascular damage not yet evident at this early stage of life.

## Acknowledgments

We are extremely grateful to all the families who took part in this study, the midwives for their help in recruiting them, and the whole ALSPAC team, which includes interviewers, computer and laboratory technicians, clerical workers, research scientists, volunteers, managers, receptionists, and nurses. We also thank Frank Dudbridge for ongoing conversations regarding genetic risk scores used in Mendelian randomization methodologies.

## Sources of Funding

Professor Timpson is a Wellcome Trust Investigator (202802/Z/16/Z), is a program lead in the Medical Research Council Integrative Epidemiology Unit (MC_UU_12013/3), and works within the University of Bristol National Institute for Health Research Biomedical Research Center. The UK Medical Research Council and the Wellcome Trust (102215/2/13/2) and the University of Bristol provide core support for ALSPAC. This publication is the work of the authors, and Dr Wade and Professor Timpson serve as guarantors for the contents of this paper. This research was specifically funded through grants from the British Heart Foundation (RG/10/004/28240, PG/06/145, and CS/15/6/31468) and the UK Medical Research Council, University of Bristol, and Wellcome Trust (MC_UU_12013/1–9 and 096989/Z11/Z to Dr Wade, 086676/7/08/Z to Professor Hughes and MR/M009351/1 to Dr Fraser, and MR/M020894/1 to Dr Howe). GWAS data were generated by Sample Logistics and Genotyping facilities at Wellcome Sanger Institute and LabCorp (Laboratory Corporation of America) using support from 23andMe.

## Disclosures

None.

## Supplementary Material
