# Supplementary material for: Assessing the Causal Role of Body Mass Index on Cardiovascular Health in Young Adults: Mendelian Randomization and Recall-by-Genotype Analyses
Source: Circulation. 2018 Nov 12;138(20):2187–201. doi: 10.1161/CIRCULATIONAHA.117.033278 (PMC6250296; doi:10.1161/CIRCULATIONAHA.117.033278)
Supplement: Supplementary file 1 [file cir-138-2187-s001.pdf]

SUPPLEMENTARY MATERIAL

SUPPLEMENTARY METHODS

Cardiovascular phenotypic measurement at age 17

The following cardiovascular phenotypes were used in MR analyses at age 17.

Blood pressure and heart rate

Sitting BP and heart rate were measured in both arms with an Omron 705 IT oscillometric BP monitor in accordance with European Society of Hypertension guidelines.[1] The average of the final two of three readings was used and the arm with the greatest number of valid observations was used for analyses. From the systolic and diastolic BP readings (SBP and DBP, respectively), mean arterial pressure (MAP) was calculated (DBP + (SBP-DBP)/3). Pulse pressure (PP) was calculated as the difference between SBP and DBP.

Pulse-wave velocity (PWV)

Aortic stiffness (carotid-femoral PWV) was assessed using a Vicorder device (Skidmore Medical, UK). Participants rested supine on a couch with their head raised to 30°. Real-time pulse-wave measures were recorded between proximal (right carotid) and distal (the upper right thigh) sensor cuffs and with the time delay between the two simultaneously measured cardiac cycles measured. Transit distance was measured from suprasternal notch directly to the top of the thigh cuff. Measurements were taken until pressure waveforms over the carotid and thigh area were of high quality and reproducible. Three carotid-femoral PWV measurements, within ≤0.5 m/s of each other, were averaged.

Carotid intima-media thickness (cIMT)

Common carotid artery B-mode ultrasound images were acquired in the ear-to-ear plane with the head rotated to 45° from the midpoint using a Zonare Z.OneUltra system equipped with a L10-5 linear transducer (Zonare Medical Systems, CA, US). Images were recorded in Digital

Imaging and Communications in Medicine (DICOM) format as 10 second cine-loop files for offline analysis using the Carotid Analyzer (Medical Imaging Applications, Coralville, IA). Left and right cIMT were taken to be the average of three end-diastolic measurements located on the far-wall of a single segment of arterial wall 5–10 mm in length and 10 mm proximal to the bifurcation. The mean of left and right cIMT was calculated and used in analyses.

Left ventricular mass (LVM)

A sub-sample of study participants from the 17-year clinic underwent echocardiography using a HDI 5000 ultrasound machine (Phillips) and P4-2 Phased Array ultrasound transducer using a standard examination protocol. LVM was estimated according to American Society of Echocardiography (ASE) guidelines.[2]

Cardiovascular phenotypic measurement at age 21

The following detailed cardiovascular phenotypes were also measured in the two RbG groups at age 21.

Blood pressure and heart rate

BP and heart rate were measured in the right arm in the supine position using a digital automated sphygmomanometer (Omron M6, Omron Healthcare, Netherlands) and the mean of two values used for analyses. MAP and PP were calculated in the same way as at 17 years.

Pulse-wave velocity

Arterial stiffness was assessed using applanation tonometry (SphygmoCor Vx, AtCor Medical, NSW, Australia), with PWV estimated using ECG-gated pulse waves travelling between carotid-femoral sites. The patient was rested in a supine position and a handheld tonometer was placed over the left carotid artery in order to allow the recording of 10-12 clear and reproducible pressure waveforms. The same tonometer was then used to measure a similar number of femoral

arterial pulse waveforms in the inguinal crease at the top of the right leg. Transit distance was measured between the upper edge of the suprasternal notch and the femoral pulse measurement site via the umbilicus using a tape measure. The device software calculated the mean transit time (in milliseconds) from the recorded pulse waveforms and carotid-femoral PWV was calculated as the transit distance/transit time.

Carotid intima-media thickness

Ultrasound assessment of the thickness of intima media interface was measured using an identical protocol to that described for age 17.

Cardiovascular Magnetic Resonance Imaging (MRI)

All measures were made using a 1.5T MR scanner (Avanto, Siemens Medical Solutions, Erlangen, Germany). Endocardial borders of the left ventricle (LV) were traced manually on short axis stacks at end-diastole and end-systole to evaluate end-diastolic volume (EDV) and end-systolic volume (ESV). Stroke volume (SV) was obtained by subtracting ESV from EDV. Epicardial borders were traced in end-diastole to calculate an epicardial volume. The EDV was subtracted from this volume, multiplied by assumed myocardial density to obtain LVM.

Flow quantification was performed through-plane in a cross-section of the ascending aorta as it passes the bifurcation of the pulmonary arteries using an ECG-gated spiral phase-contrast MRI sequence, as described previously.[3] This technique allows images to be acquired within a short breath-hold (0.5 seconds) with a spatial resolution of 1.6 x 1.6 mm and a temporal resolution of 30 milliseconds. All images were processed using in-house plug-ins for the Open source software OsiriX (OsiriX Foundation, Geneva, Switzerland). Flow images were manually segmented (using the modulus images) and SV (ml) was measured and cardiac output (CO, L/min) was calculated as SV x heart rate. At the time of flow imaging, BP was simultaneously measured using MRI-compatible oscillometric sphygmomanometer (Datex Ohmeda). Systemic vascular resistance (SVR; measured in mmHg/L/min) was calculated by dividing the measured

mean BP by CO. Total arterial compliance (TAC) was calculated by optimisation of the two-element Windkessel model, as previously described.[4] Briefly, the flow curves and SVR were used as inputs to the model. PP was calculated for a series of modelled pressure curves generated using a range of TAC values from 0.1 to 5.0 mL/mmHg in increments of 0.01. The compliance value that gave the smallest error between the modelled PP and the true PP was taken to be the true compliance.

Genotyping

Participants were genotyped using the Illumina HumanHap550 quad genome-wide SNP genotyping platform.[5] Participants were excluded due to having at least one of: incorrectly recorded sex, minimal or excessive heterozygosity, disproportionate levels of individual missingness, evidence of cryptic relatedness or non-European ancestry.[5] SNPs with a minor allele frequency (MAF) of <1% and call rate of <95% were removed and only SNPs that passed an exact test of Hardy-Weinberg equilibrium (P<5x10^{-7}) were included. For this project and at the time of recall, imputation of genotypes was conducted with MACH 1.0.16 Markov Chain Haplotyping software, using CEPH individuals from phase 2 of the HapMap project as a reference (release #22), where imputation quality was high (>0.9).

Confounders

At enrolment, mothers reported their educational attainment and both her and her partner's occupation. Highest household occupation was used to assign participants a household social class, using the 1991 British Office of Population Census Statistics classification.[6] Both maternal education and household social class were used as a general indication of socioeconomic position.

Smoking status was obtained by questionnaire and participants were coded as "ever" or "never" smokers. The most recent record of physical activity was defined as the counts per minute (CPM) and minutes spent in moderate-to-vigorous activity (MVPA) obtained from a subsample of

participants at the 15-year clinic by an MTI Actigraph AM7164 2.2 accelerometer worn for 7 days.[7] Additionally, the most recent measurement of dietary intake of the participant was total energy intake at age 13, previously estimated from linear spline multi-level models of the combination of food frequency questionnaires and diet diaries.[8]

For analyses using data from the 21-year RbG group, smoking status and physical activity were obtained via questionnaire at the time of cardiovascular phenotyping; however, information on recent dietary intake was not available. Participants at this age were similarly classed as "ever" or "never" smokers and weekly exercise was categorised as either "never/rarely/<2 times a week" or "≥2 times a week".

Recall-by-genotype power calculation, assembly and sampling method

Original beta-coefficients for the genome-wide genetic risk score (GRS) assembly were obtained directly from the genome-wide association study (GWAS) of body mass index (BMI), conducted in 2010 by Speliotes *et al*.,[9] with the Avon Longitudinal Study of Parents and Children (ALSPAC) removed from the release. All alleles were aligned such that the reference allele matched the BMI-increasing direction reported in the GWAS and PLINK (--score) was used to derive a score for each participant (N=8,350). After removing heterozygous haploid genotypes (N=46,067), direct genotypes were available for 500,527 SNPs, of which 472,208 mapped to SNPs that overlapped with the Speliotes *et al.* GWAS results and 470,667 mapped to alleles. The ALSPAC genetic dataset used was the best available data at the time of the recall-by-genotype (RbG) study design (2012) and was subsequently used in several papers.[10,11]

The number of participants invited and recruited to the RbG study were orchestrated based on an a priori power calculation taking into account the predicted explained variation in BMI by the genome-wide GRS and its effect size on an example cardiovascular phenotype (SBP) Specifically, using the observed association between SBP and BMI at age 17, we calculated that we would require 450 participants in our RbG to be able to detect a difference of 3mmHg (a difference that in young adulthood has clinically meaningful association with future CVD[12]) with

80% power and a two-sided p-value threshold of 0.05, and assuming an approximate 3.3kg/m^2 unit difference in BMI between recalled groups.

Participants were then recruited according to their appearance in nine sampling groups, from the most extreme to the least, to maximise power and difference in BMI. These samples were as follows (see figure below):

- 3%, 6%, 9%, 12%, 15%, 18%, 21%, 24%, 27% from the lower tail of the genome-wide GRS distribution
- 97%, 94%, 91%, 88%, 85%, 82%, 79%, 76%, 73% from the upper tail of the genome-wide GRS distribution

The GRS was computed for 4,602 individuals in total within these sampling groups. Of those who were invited to the study (N=2,071), 419 individuals attended from across the entire distribution of sampling groups (see Supporting Figures below) and had both full genetic and BMI data.

Supporting Figure 1. Participants invited to the RbG study based on sampling groups across the lower and upper 30% of a genome-wide GRS distribution

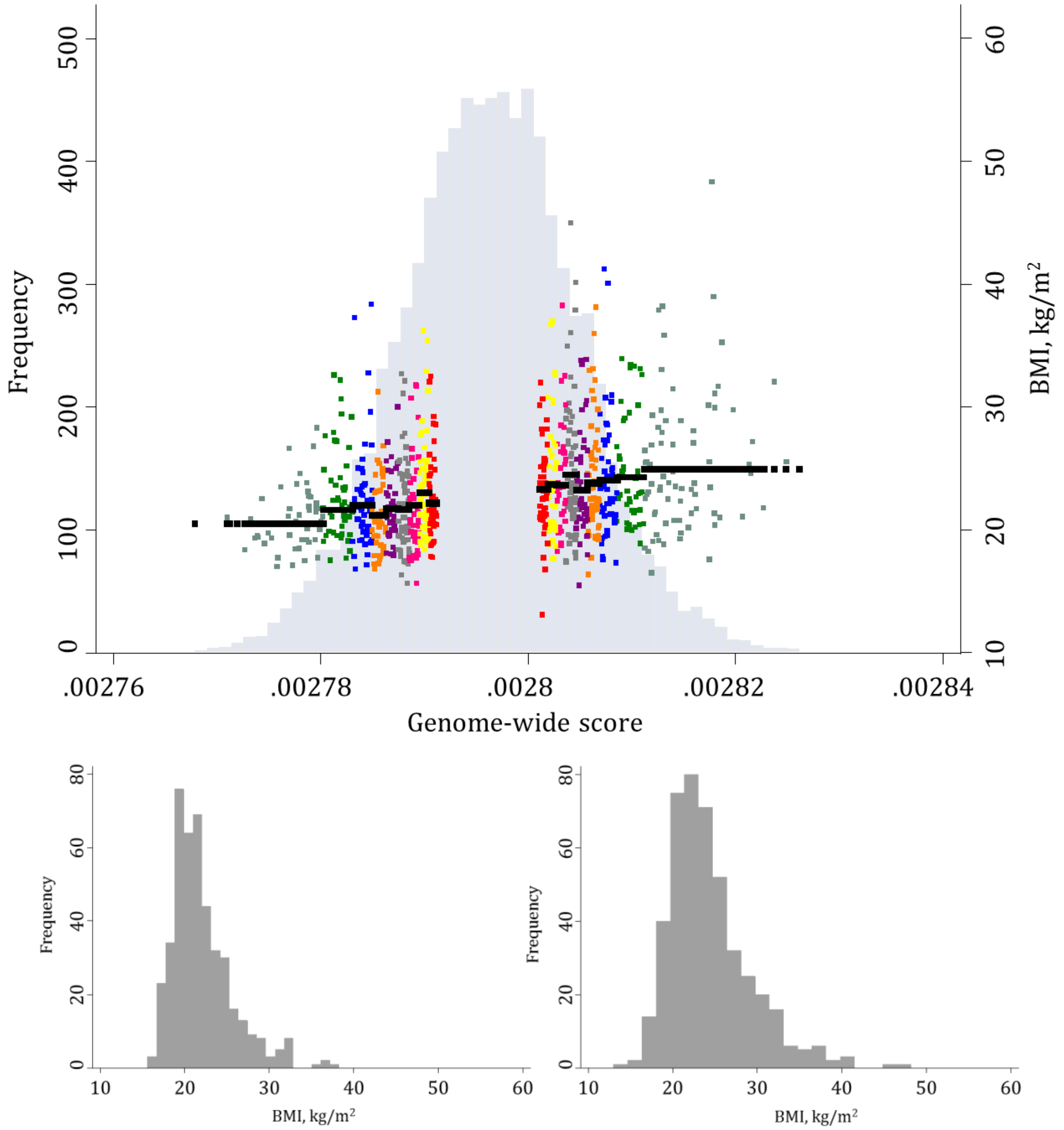

The light grey histogram (top panel) represents the genome-wide GRS distribution (frequency, left-hand axes). The overlaying scatter graph represents the corresponding values of BMI at age 17 (kg/m², right-hand axis) from everyone invited with each value of the genome-wide GRS (N=2,071). Each colour represents the sampling groups from which participants were invited. The black line represents the mean BMI values within each sampling group. The dark grey histograms show the distribution of BMI at age 17 (kg/m²) in those invited from the lower (bottom left panel) and upper (bottom right panel) tails of the genome-wide GRS distribution.

Supporting Figure 2. Participants who attended the RbG study based on sampling groups across the lower and upper 30% of a genome-wide GRS distribution

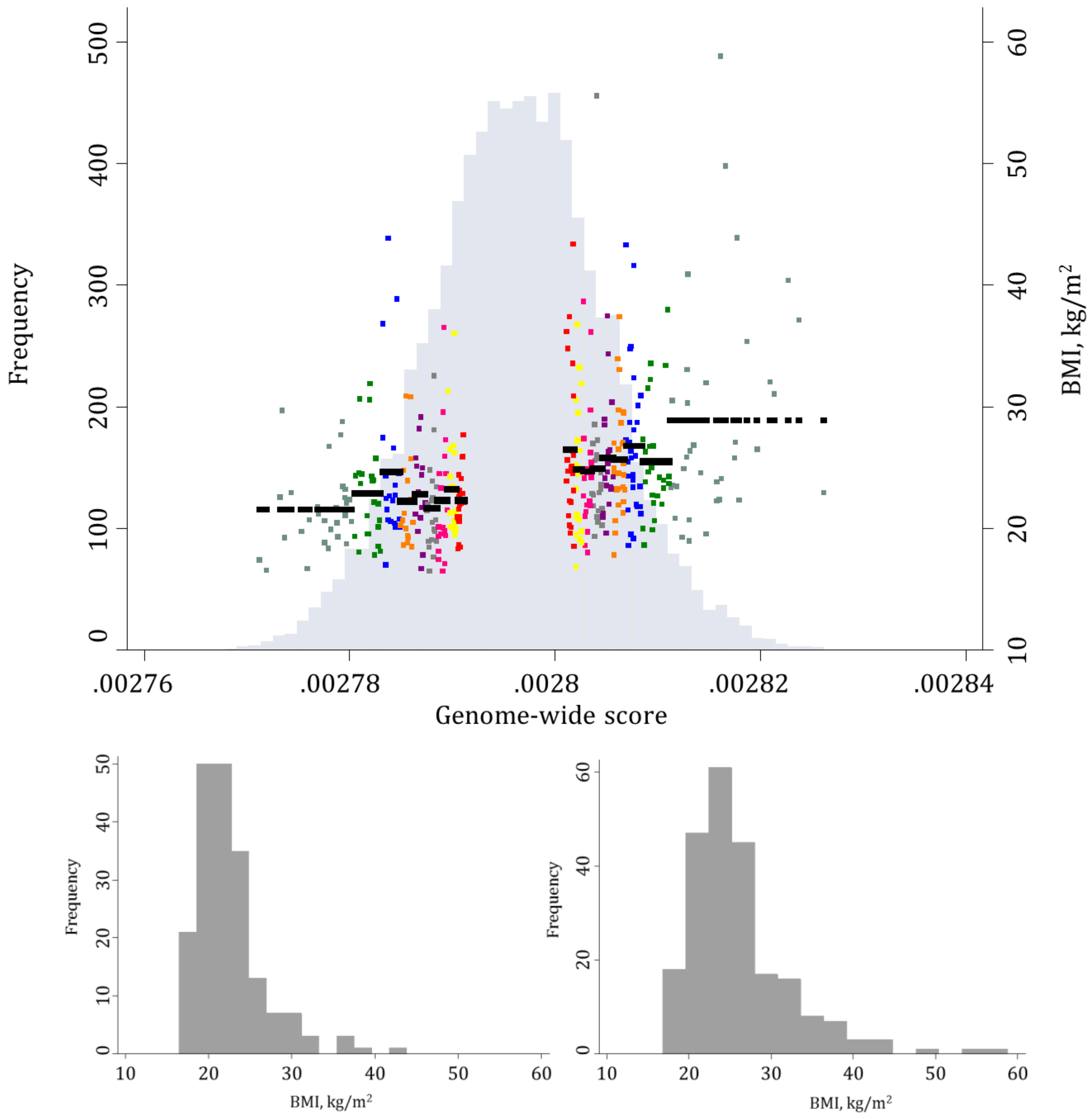

The light grey histogram (top panel) represents the genome-wide GRS distribution (frequency, left-hand axes). The overlaying scatter graph represents the corresponding values of BMI at age 21 (kg/m², right-hand axis) from everyone included in the study with each value of the genome-wide GRS (N=419). Each colour represents the sampling groups from which participants were invited. The black line represents the mean BMI values within each sampling group. The dark grey histograms show the distribution of BMI at age 21 (kg/m²) in those included from the lower (bottom left panel) and upper (bottom right panel) tails of the genome-wide GRS distribution.

Supporting Figure 3. Comparison of BMI at age 17 and the genome-wide GRS between those who participated in the RbG study and those who were eligible for the RbG study but did not participate.

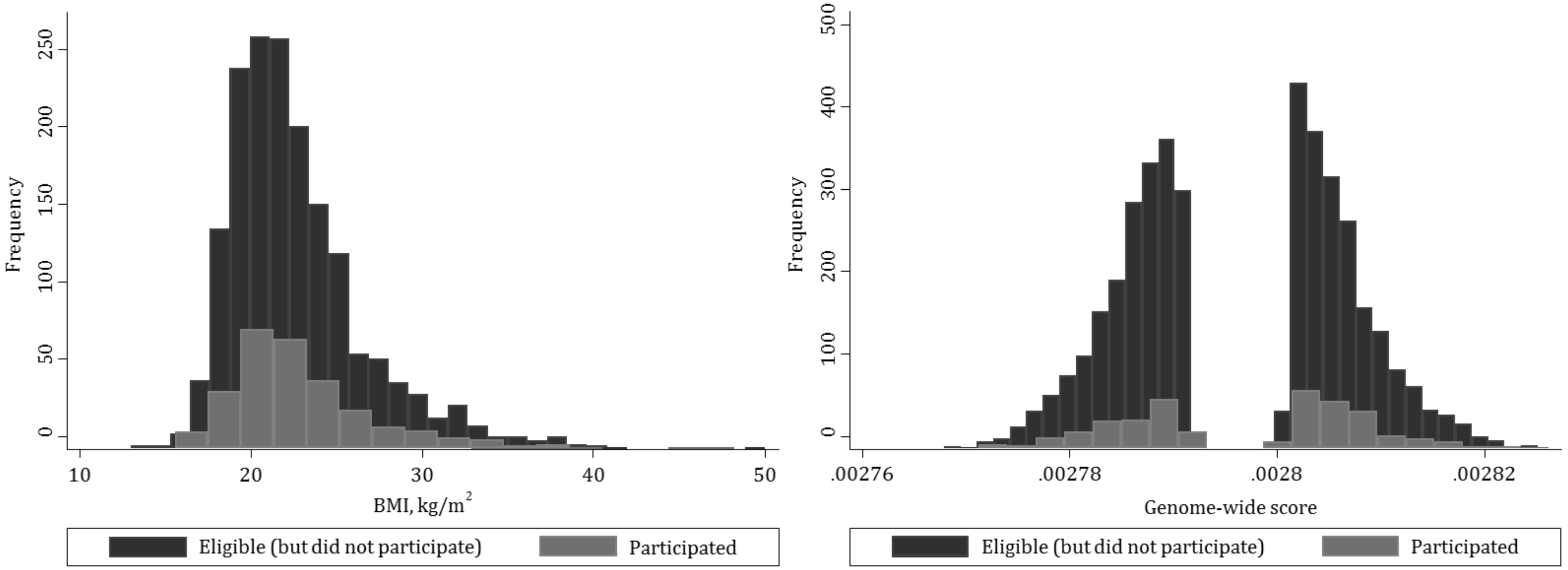

Variable	Mean (SD), N		*P*-value for difference between those who invited and participated*
	Eligible (but did not participate)	Participated in the RbG study	
BMI at age 17 (kg/m^2)	22.68 (4.05), 1765	23.15 (4.62), 312	0.07
Genome-wide GRS	0.003 (0.00001), 4183	0.003 (0.00001), 419	0.16

**Obtained by t-test statistic*

SUPPLEMENTARY TABLES

Supplementary Table S1. Descriptive statistics for ALSPAC 17-year clinic comparing those who had complete vs. any missing data on all variables included in multivariable regression analyses

Variable	N	Mean (SD) or percentage in those with complete data	N	Mean (SD) or percentage in those with missing data
Participant's phenotypes				
Age (years)	498	17.66 (0.01)	2995	17.81 (0.01)
Sex (% female)	498	53.61	7411	47.42
BMI (kg/m^2)	498	22.60 (0.16)	2906	22.75 (0.08)
SBP (mmHg)	498	119.27 (0.48)	2674	118.49 (0.21)
DBP (mmHg)	498	63.08 (0.26)	2674	63.75 (0.13)
PP (mmHg)	498	56.19 (0.45)	2674	54.73 (0.19)
MAP (mmHg)	498	81.81 (0.28)	2674	82.00 (0.13)
Mean cIMT (mm)	498	0.48 (0.002)	2645	0.48 (0.001)
Carotid-femoral PWV (m/s)	498	5.80 (0.03)	2031	5.74 (0.02)
LVMI (g/m$^{2.7}$)	498	29.37 (0.28)	922	28.68 (0.20)
Heart rate (bpm)	498	63.48 (0.43)	2674	64.64 (0.19)
Smoking status (% ever smoked)	498	40.36	2346	53.92
Physical activity at age 15				
CPM (counts)	498	488.08 (8.27)	1189	482.43 (5.21)
MVPA (minutes)	498	24.81 (0.88)	1189	23.28 (0.53)
Dietary intake (kcal)	498	2255.79 (8.51)	6643	2260.58 (2.26)
BMI GRS (comprising 97 SNPs)	498	87.97 (0.27)	7411	88.37 (0.07)
Parental phenotypes				
Highest household social class	498		6100	
I	106	21.29	841	13.79
II	240	48.19	2662	43.64
III (non-manual)	97	19.48	1554	25.48
III (manual)	34	6.83	735	12.05
IV	20	4.02	270	4.43
V	1	0.20	38	0.62
Maternal education	498		6484	
CSE	43	8.63	1113	17.17
Vocational	34	6.83	601	9.27
O-Level	162	32.53	2291	35.33
A-Level	147	29.52	1553	23.95
Degree	112	22.49	926	14.28

ALSPAC = Avon Longitudinal Study of Parents and Children; BMI = body mass index; cIMT = carotid intima-media thickness; CPM = counts per minute; CSE = certificate of secondary education; DBP = diastolic blood pressure; GRS = genetic risk score; LVMI = left ventricular mass indexed to height$^{2.7}$; MAP = mean arterial pressure; MVPA = minutes spent in moderate-to-vigorous activity; PP = pulse pressure; PWV = pulse wave velocity; SBP = systolic blood pressure; SD = standard deviation; SNP = single nucleotide polymorphism.

Supplementary Table S2. Associations between confounders and BMI in ALSPAC 17-year clinic

**Confounder**	**N**	**Difference in mean BMI (kg/m^2) with each confounder (95% CI)**	***P*-value**
Highest household social class	3175		
I		reference	
II		0.60 (0.21, 0.98)	0.002
III (non-manual)		0.85 (0.41, 1.29)	0.0001
III (manual)		1.16 (0.59, 1.73)	6.85x10^{-05}
IV		1.35 (0.53, 2.17)	0.001
V		3.25 (1.25, 5.25)	0.001
Maternal education	3058		
CSE		reference	
Vocational		-0.73 (-1.40, -0.06)	0.03
O-Level		-0.72 (-1.20, -0.24)	0.003
A-Level		-1.05 (-1.54, -0.56)	3.14x10^{-05}
Degree		-1.71 (-2.23, -1.19)	1.40x10^{-10}
Gender	3404		
Male		reference	
Female		0.23 (-0.04, 0.50)	0.09
Age at 17 (years)	3404	0.70 (0.38, 1.02)	2.36x10^{-05}
Smoking status	3805		
Never		reference	
Ever		0.50 (0.21, 0.79)	0.001
Physical activity			
CPM (counts)	1356	-0.0005 (-0.002, 0.001)	0.43
MVPA (minutes)	1356	-0.01 (-0.02, 0.004)	0.22
Dietary intake (kcal)	3361	0.003 (0.002, 0.004)	2.66x10^{-18}

ALSPAC = Avon Longitudinal Study of Parents and Children; BMI = body mass index; CI = confidence interval; CPM = counts per minute; CSE = certificate of secondary education; MVPA = minutes spent in moderate-to-vigorous physical activity

Estimates represent the difference in BMI (kg/m^2) per unit increase in each continuous confounder or level increase in each categorical or binary confounder

Supplementary Table S3. Associations between confounders and SBP in ALSPAC 17-year clinic

**Confounder**	**N**	**Difference in mean SBP (mmHg) with each confounder (95% CI)**	***P*-value**
Highest household social class	2864		
I		reference	
II		0.89 (-0.22, 1.99)	0.12
III (non-manual)		1.05 (-0.22, 2.32)	0.11
III (manual)		1.80 (0.14, 3.46)	0.03
IV		0.49 (-1.89, 2.87)	0.69
V		-0.86 (-6.46, 4.75)	0.76
Maternal education	2965		
CSE		reference	
Vocational		-0.19 (-2.14, 1.75)	0.84
O-Level		-1.39 (-2.79, 0.01)	0.05
A-Level		-1.37 (-2.80, 0.06)	0.06
Degree		-2.08 (-3.59, -0.58)	0.01
Gender	3170		
Male		reference	
Female		-10.65 (-11.32, -9.98)	5.07×10^{-213}
Age at 17 (years)	3172	-0.63 (-1.67, 0.41)	0.24
Smoking status	2689		
Never		reference	
Ever		-0.48 (-1.31, 0.35)	0.25
Physical activity			
CPM (counts)	1298	0.01 (0.01, 0.01)	5.93×10^{-09}
MVPA (minutes)	1298	0.08 (0.05, 0.11)	5.31×10^{-07}
Dietary intake (kcal)	3140	0.03 (0.03, 0.03)	4.17×10^{-214}

ALSPAC = Avon Longitudinal Study of Parents and Children; BMI = body mass index; CI = confidence interval; CPM = counts per minute; CSE = certificate of secondary education; MVPA = minutes spent in moderate-to-vigorous physical activity; SBP = systolic blood pressure
Estimates represent the difference in SBP (mmHg) per unit increase in each continuous confounder or level increase in each categorical or binary confounder

Supplementary Table S4. Associations between confounders and DBP in ALSPAC 17-year clinic

**Confounder**	**N**	**Difference in mean DBP (mmHg) with each confounder (95% CI)**	***P*-value**
Highest household social class	2864		
I		reference	
II		0.93 (0.27, 1.60)	0.01
III (non-manual)		1.31 (0.54, 2.07)	0.001
III (manual)		2.46 (1.46, 3.45)	$1.33x10^{-06}$
IV		1.00 (-0.43, 2.42)	0.17
V		1.40 (-1.96, 4.75)	0.41
Maternal education	2965		
CSE		reference	
Vocational		-1.70 (-2.87, -0.54)	0.004
O-Level		-1.52 (-2.36, -0.68)	0.0004
A-Level		-2.12 (-2.98, -1.26)	$1.31x10^{-06}$
Degree		-2.86 (-3.76, -1.96)	$5.83x10^{-10}$
Gender	3172		
Male		reference	
Female		1.20 (0.74, 1.65)	$2.90x10^{-07}$
Age at 17 (years)	3172	1.65 (1.03, 2.28)	$1.83x10^{-07}$
Smoking status	2689		
Never		reference	
Ever		0.60 (0.11, 1.10)	0.02
Physical activity			
CPM (counts)	1298	-0.003 (-0.01, -0.001)	0.002
MVPA (minutes)	1298	-0.03 (-0.05, -0.01)	0.001
Dietary intake (kcal)	3140	-0.001 (-0.002, 0.0003)	0.15

ALSPAC = Avon Longitudinal Study of Parents and Children; BMI = body mass index; CI = confidence interval; CPM = counts per minute; CSE = certificate of secondary education; DBP = diastolic blood pressure; MVPA = minutes spent in moderate-to-vigorous physical activity
Estimates represent the difference in DBP (mmHg) per unit increase in each continuous confounder or level increase in each categorical or binary confounder

Supplementary Table S5. Associations between confounders and PP in ALSPAC 17-year clinic

**Confounder**	**N**	**Difference in mean PP (mmHg) with each confounder (95% CI)**	***P*-value**
Highest household social class	2864		
I		reference	
II		-0.05 (-1.07, 0.97)	0.93
III (non-manual)		-0.26 (-1.42, 0.91)	0.67
III (manual)		-0.66 (-2.18, 0.87)	0.40
IV		-0.50 (-2.69, 1.68)	0.65
V		-2.26 (-7.41, 2.89)	0.39
Maternal education	2965		
CSE		reference	
Vocational		1.51 (-0.27, 3.29)	0.10
O-Level		0.13 (-1.16, 1.41)	0.85
A-Level		0.75 (-0.56, 2.07)	0.26
Degree		0.78 (-0.60, 2.16)	0.27
Gender	3172		
Male		reference	
Female		-11.84 (-12.42, -11.27)	$<4.51 \times 10^{-308}$
Age at 17 (years)	3189	-2.28 (-3.24, -1.33)	2.92×10^{-06}
Smoking status	2689		
Never		reference	
Ever		-1.08 (-1.84, -0.32)	0.01
Physical activity			
CPM (counts)	1298	0.01 (0.01, 0.02)	1.24×10^{-16}
MVPA (minutes)	1298	0.11 (0.08, 0.14)	8.23×10^{-14}
Dietary intake (kcal)	3140	0.03 (0.03, 0.03)	3.32×10^{-291}

ALSPAC = Avon Longitudinal Study of Parents and Children; BMI = body mass index; CI = confidence interval; CPM = counts per minute; CSE = certificate of secondary education; MVPA = minutes spent in moderate-to-vigorous physical activity; PP = pulse pressure
Estimates represent the difference in PP (mmHg) per unit increase in each continuous confounder or level increase in each categorical or binary confounder

Supplementary Table S6. Associations between confounders and MAP in ALSPAC 17-year clinic

**Confounder**	**N**	**Difference in mean MAP (mmHg) with each confounder (95% CI)**	***P*-value**
Highest household social class	2864		
I		reference	
II		0.92 (0.23, 1.61)	0.01
III (non-manual)		1.22 (0.43, 2.01)	0.002
III (manual)		2.24 (1.21, 3.27)	0.00002
IV		0.83 (-0.65, 2.30)	0.27
V		0.65 (-2.83, 4.12)	0.72
Maternal education	2965		
CSE		reference	
Vocational		-1.20 (-2.41, 0.005)	0.05
O-Level		-1.48 (-2.34, -0.61)	0.001
A-Level		-1.87 (-2.76, -0.98)	0.00004
Degree		-2.60 (-3.54, -1.67)	$5.26x10^{-08}$
Gender			
Male	3172	reference	
Female		-2.75 (-3.22, -2.29)	$1.58x10^{-30}$
Age at 17 (years)	3172	0.89 (0.25, 1.54)	0.01
Smoking status	2689		
Never		reference	
Ever		0.24 (-0.27, 0.75)	0.36
Physical activity			
CPM (counts)	1298	0.001 (-0.001, 0.003)	0.27
MVPA (minutes)	1298	0.01 (-0.01, 0.03)	0.54
Dietary intake (kcal)	3140	0.01 (0.01, 0.01)	$7.94x10^{-46}$

ALSPAC = Avon Longitudinal Study of Parents and Children; BMI = body mass index; CI = confidence interval; CPM = counts per minute; CSE = certificate of secondary education; MAP = mean arterial pressure; MVPA = minutes spent in moderate-to-vigorous physical activity
Estimates represent the difference in MAP (mmHg) per unit increase in each continuous confounder or level increase in each categorical or binary confounder

Supplementary Table S7. Associations between confounders and mean cIMT in ALSPAC 17-year clinic

**Confounder**	**N**	**Difference in mean cIMT (mm) with each confounder (95% CI)**	***P*-value**
Highest household social class	2838		
I		reference	
II		0.0001 (-0.004, 0.005)	0.95
III (non-manual)		0.0002 (-0.005, 0.01)	0.93
III (manual)		-0.003 (-0.01, 0.004)	0.38
IV		-0.01 (-0.02, 0.003)	0.17
V		-0.01 (-0.04, 0.01)	0.30
Maternal education	2937		
CSE		reference	
Vocational		0.005 (-0.003, 0.01)	0.22
O-Level		0.003 (-0.002, 0.01)	0.26
A-Level		0.001 (-0.01, 0.01)	0.82
Degree		0.01 (-0.0003, 0.01)	0.06
Gender	3143		
Male		reference	
Female		-0.01 (-0.02, -0.01)	3.97×10^{-19}
Age at 17 (years)	3142	0.0001 (-0.004, 0.004)	0.96
Smoking status	2664		
Never		reference	
Ever		-0.001 (-0.004, 0.003)	0.67
Physical activity			
CPM (counts)	1292	0.00002 (0.000002, 0.00003)	0.03
MVPA (minutes)	1292	0.0001 (-0.00001, 0.0002)	0.08
Dietary intake (kcal)	3110	0.00003 (0.00002, 0.00004)	3.30×10^{-15}

ALSPAC = Avon Longitudinal Study of Parents and Children; BMI = body mass index; CI = confidence interval; cIMT = carotid intima media thickness; CSE = certificate of secondary education; MVPA = minutes spent in moderate-to-vigorous physical activity
Estimates represent the difference in mean cIMT (mm) per unit increase in each continuous confounder or level increase in each categorical or binary confounder

Supplementary Table S8. Associations between confounders and carotid-femoral PWV in ALSPAC 17-year clinic

**Confounder**	**N**	**Difference in PWV (%) with each confounder (95% CI)***	***P*-value**
Highest household social class	2289		
I		reference	
II		-0.54 (-1.81, 0.74)	0.41
III (non-manual)		-0.95 (-2.39, 0.51)	0.20
III (manual)		0.21 (-1.70, 2.16)	0.83
IV		-0.91 (-3.66, 1.92)	0.52
V		3.97 (-3.21, 11.68)	0.29
Maternal education	2365		
CSE		reference	
Vocational		-0.86 (-3.13, 1.45)	0.46
O-Level		-1.50 (-3.12, 0.15)	0.08
A-Level		-1.88 (-3.52, -0.20)	0.03
Degree		-1.98 (-3.71, -0.22)	0.03
Gender	2529		
Male		reference	
Female		-8.30 (-9.07, -7.53)	5.33×10^{-91}
Age at 17 (years)	2529	2.25 (0.81, 3.71)	0.002
Smoking status	2193		
Never		reference	
Ever		-0.38 (-1.35, 0.59)	0.44
Physical activity			
CPM (counts)	1069	0.01 (0.003, 0.01)	0.0004
MVPA (minutes)	1069	0.09 (0.05, 0.13)	3.33×10^{-06}
Dietary intake (kcal)	2507	0.02 (0.02, 0.02)	3.44×10^{-68}

ALSPAC = Avon Longitudinal Study of Parents and Children; BMI = body mass index; CI = confidence interval; CPM = counts per minute; CSE = certificate of secondary education; MVPA = minutes spent in moderate-to-vigorous physical activity; PWV = pulse wave velocity
**Percentage change in PWV (m/s) per unit increase in each continuous confounder or level increase in each categorical or binary confounder. For analyses, carotid-femoral PWV was log-transformed for normalization of the residuals; therefore, differences and confidence intervals were back-transformed and represent the mean percentage (%) difference in PWV.*

Supplementary Table S9. Associations between confounders and LVMI in ALSPAC 17-year clinic

**Confounder**	**N**	**Difference in mean LVMI ($g/m^{2.7}$) with each confounder (95% CI)**	***P*-value**
Highest household social class	1291		
I		reference	
II		0.10 (-0.79, 0.99)	0.83
III (non-manual)		0.84 (-0.20, 1.88)	0.11
III (manual)		0.48 (-0.90, 1.87)	0.49
IV		-0.49 (-2.40, 1.42)	0.61
V		-5.90 (-12.91, 1.11)	0.10
Maternal education	1333		
CSE		reference	
Vocational		0.29 (-1.28, 1.86)	0.72
O-Level		0.44 (-0.73, 1.61)	0.46
A-Level		-0.37 (-1.58, 0.83)	0.54
Degree		-0.54 (-1.79, 0.72)	0.40
Gender	1420		
Male		reference	
Female		-2.36 (-2.99, -1.73)	2.65×10^{-13}
Age at 17 (years)	1420	0.25 (-0.76, 1.25)	0.63
Smoking status	1265		
Never		reference	
Ever		0.17 (-0.51, 0.84)	0.63
Physical activity			
CPM (counts)	682	0.003 (0.0003, 0.01)	0.03
MVPA (minutes)	682	0.03 (0.01, 0.06)	0.004
Dietary intake (kcal)	1407	0.01 (0.01, 0.01)	6.04×10^{-18}

ALSPAC = Avon Longitudinal Study of Parents and Children; BMI = body mass index; CI = confidence interval; CPM = counts per minute; CSE = certificate of secondary education; LVMI = left ventricular mass indexed to height$^{2.7}$; MVPA = minutes spent in moderate-to-vigorous physical activity

Estimates represent the difference in LVMI ($g/m^{2.7}$) per unit increase in each continuous confounder or level increase in each categorical or binary confounder

Supplementary Table S10. Associations between confounders and heart rate in ALSPAC 17-year clinic

**Confounder**	**N**	**Difference in mean heart rate (bpm) with each confounder (95% CI)**	***P*-value**
Highest household social class	2864		
I		reference	
II		1.52 (0.53, 2.50)	0.003
III (non-manual)		2.00 (0.87, 3.14)	0.001
III (manual)		1.83 (0.35, 3.32)	0.02
IV		3.40 (1.28, 5.53)	0.002
V		3.08 (-1.92, 8.09)	0.23
Maternal education	2965		
CSE		reference	
Vocational		-2.74 (-4.48, -1.00)	0.002
O-Level		-1.62 (-2.88, -0.37)	0.01
A-Level		-2.41 (-3.70, -1.13)	0.0002
Degree		-3.00 (-4.35, -1.66)	0.00001
Gender	3172		
Male		reference	
Female		4.39 (3.72, 5.06)	5.37×10^{-37}
Age at 17 (years)	3172	0.79 (-0.14, 1.72)	0.10
Smoking status	2689		
Never		reference	
Ever		0.35 (-0.38, 1.08)	0.35
Physical activity			
CPM (counts)	1298	-0.01 (-0.01, -0.005)	1.01×10^{-07}
MVPA (minutes)	1298	-0.06 (-0.09, -0.04)	6.09×10^{-06}
Dietary intake (kcal)	3140	-0.01 (-0.01, -0.01)	7.83×10^{-30}

ALSPAC = Avon Longitudinal Study of Parents and Children; BMI = body mass index; CI = confidence interval; CSE = certificate of secondary education; MVPA = minutes spent in moderate-to-vigorous physical activity

Estimates represent the difference in heart rate (bpm) per unit increase in each continuous confounder or level increase in each categorical or binary confounder

Supplementary Table S11. Associations between confounders and the weighted GRS (comprising 97 SNPs) in ALSPAC 17-year clinic

**Confounder**	**N**	**Difference in the weighted GRS with each confounder (95% CI)**	***P*-value**
Highest household social class	6598		
I		reference	
II		0.16 (-0.30, 0.61)	0.50
III (non-manual)		0.45 (-0.05, 0.95)	0.08
III (manual)		0.07 (-0.52, 0.67)	0.80
IV		0.01 (-0.81, 0.83)	0.98
V		3.54 (1.54, 5.53)	0.001
Maternal education	6598		
CSE		reference	
Vocational		-0.01 (-0.62, 0.59)	0.96
O-Level		-0.30 (-0.73, 0.14)	0.18
A-Level		-0.15 (-0.62, 0.31)	0.52
Degree		-0.39 (-0.91, 0.13)	0.14
Gender	7909		
Male		reference	
Female		0.02 (-0.25, 0.29)	0.89
Age at 17 (years)	3493	0.25 (-0.24, 0.73)	0.32
Smoking status	2844		
Never		reference	
Ever		0.20 (-0.26, 0.66)	0.39
Physical activity			
CPM (counts)	1687	-0.0001 (-0.002, 0.002)	0.94
MVPA (minutes)	1687	-0.001 (-0.02, 0.01)	0.87
Dietary intake (kcal)	7141	0.002 (0.001, 0.002)	2.34×10^{-05}

ALSPAC = Avon Longitudinal Study of Parents and Children; BMI = body mass index; CI = confidence interval; CPM = counts per minute; CSE = certificate of secondary education; GRS = genetic risk score; MVPA = minutes spent in moderate-to-vigorous physical activity; SNP = single nucleotide polymorphism

Estimates represent the difference in the GRS (comprising 97 SNPs) per unit increase in each continuous confounder or level increase in each categorical or binary confounder

Supplementary Table S12. Associations between confounders and BMI in ALSPAC 21-year RbG group

**Confounder**	**N**	**Difference in mean BMI (kg/m^2) with each confounder (95% CI)**	***P*-value**
Highest household social class	370		
I		reference	
II		0.84 (-0.57, 2.24)	0.24
III (non-manual)		2.92 (1.24, 4.61)	0.001
II (manual)		4.24 (1.87, 6.61)	0.0005
IV		5.17 (0.92, 9.41)	0.02
Maternal education	380		
CSE		reference	
Vocational		1.35 (-1.56, 4.25)	0.36
O-Level		-0.23 (-2.50, 2.05)	0.85
A-Level		-0.85 (-3.09, 1.40)	0.46
Degree		-1.57 (-3.88, 0.75)	0.18
Gender	418		
Male		reference	
Female		0.31 (-0.85, 1.47)	0.60
Age at clinic (years)	418	-0.06 (-0.64, 0.53)	0.84
Smoking status	417		
Never		reference	
Ever		1.03 (-0.42, 2.48)	0.16
Weekly exercise	299		
Never/rarely/<2 per week		reference	
≥2 times per week		0.56 (-0.74, 1.87)	0.40

ALSPAC = Avon Longitudinal Study of Parents and Children; BMI = body mass index; CI = confidence interval; CSE = certificate of secondary education; RbG = recall-by-genotype
Estimates represent the difference in BMI (kg/m^2) per unit increase in each continuous confounder or level increase in each categorical or binary confounder

Supplementary Table S13. Associations between confounders and SBP in ALSPAC 21-year RbG group

**Confounder**	**N**	**Difference in mean SBP (mmHg) with each confounder (95% CI)**	***P*-value**
Highest household social class	368		
I		reference	
II		1.52 (-1.12, 4.16)	0.26
III (non-manual)		1.21 (-1.96, 4.38)	0.45
III (manual)		3.24 (-1.21, 7.69)	0.15
IV		-0.58 (-9.14, 7.99)	0.89
Maternal education	377		
CSE		reference	
Vocational		0.05 (-5.24, 5.33)	0.99
O-Level		-2.98 (-7.13, 1.17)	0.16
A-Level		-1.52 (-5.62, 2.59)	0.47
Degree		-4.03 (-8.26, 0.21)	0.06
Gender	415		
Male		reference	
Female		9.97 (8.11, 11.83)	$3.28x10^{-23}$
Age at clinic (years)	415	0.94 (-0.12, 1.99)	0.08
Smoking status	414		
Never		reference	
Ever		1.66 (-0.99, 4.31)	0.22
Weekly exercise	298		
Never/rarely/<2 per week		reference	
≥2 times per week		3.12 (0.66, 5.58)	0.01

ALSPAC = Avon Longitudinal Study of Parents and Children; BMI = body mass index; CI = confidence interval; CSE = certificate of secondary education; RbG = recall-by-genotype; SBP = systolic blood pressure

Estimates represent the difference in SBP (mmHg) per unit increase in each continuous confounder or level increase in each categorical or binary confounder

Supplementary Table S14. Associations between confounders and DBP in ALSPAC 21-year RbG group

**Confounder**	**N**	**Difference in mean DBP (mmHg) with each confounder (95% CI)**	***P*-value**
Highest household social class	369		
I		reference	
II		1.17 (-0.54, 2.88)	0.18
III (non-manual)		0.73 (-1.32, 2.78)	0.49
III (manual)		-0.46 (-3.34, 2.43)	0.76
IV		0.95 (-4.59, 6.49)	0.74
Maternal education	378		
CSE		reference	
Vocational		-2.92 (-6.36, 0.52)	0.10
O-Level		-1.27 (-3.97, 1.43)	0.36
A-Level		-0.66 (-3.33, 2.02)	0.63
Degree		-2.01 (-4.76, 0.75)	0.15
Gender	416		
Male		reference	
Female		-1.04 (-2.39, 0.30)	0.13
Age at clinic (years)	416	-0.32 (-1.01, 0.36)	0.36
Smoking status	414		
Never		reference	
Ever		0.65 (-1.06, 2.37)	0.45
Weekly exercise	298		
Never/rarely/<2 per week		reference	
≥2 times per week		-0.78 (-2.31, 0.76)	0.32

ALSPAC = Avon Longitudinal Study of Parents and Children; BMI = body mass index; CI = confidence interval; CSE = certificate of secondary education; DBP = diastolic blood pressure
Estimates represent the difference in DBP (mmHg) per unit increase in each continuous confounder or level increase in each categorical or binary confounder

Supplementary Table S15. Associations between confounders and PP in ALSPAC 21-year RbG group

**Confounder**	**N**	**Difference in mean PP (mmHg) with each confounder (95% CI)**	***P*-value**
Highest household social class	368		
I		reference	
II		0.35 (-2.13, 2.84)	0.78
III (non-manual)		0.49 (-2.50, 3.47)	0.75
III (manual)		3.69 (-0.49, 7.88)	0.08
IV		-1.53 (-9.59, 6.53)	0.71
Maternal education	377		
CSE		reference	
Vocational		2.97 (-2.01, 7.95)	0.24
O-Level		-1.71 (-5.62, 2.20)	0.39
A-Level		-0.86 (-4.73, 3.02)	0.66
Degree		-2.02 (-6.01, 1.97)	0.32
Gender	415		
Male		reference	
Female		11.02 (9.38, 12.65)	1.07×10^{-33}
Age at 17 (years)	415	1.26 (0.28, 2.24)	0.01
Smoking status	414		
Never		reference	
Ever		1.00 (-1.47, 3.48)	0.42
Weekly exercise	298		
Never/rarely/<2 per week		reference	
≥2 times per week		3.90 (1.62, 6.18)	0.001

ALSPAC = Avon Longitudinal Study of Parents and Children; BMI = body mass index; CI = confidence interval; CSE = certificate of secondary education; PP = pulse pressure
Estimates represent the difference in PP (mmHg) per unit increase in each continuous confounder or level increase in each categorical or binary confounder

Supplementary Table S16. Associations between confounders and MAP in ALSPAC 21-year RbG group

**Confounder**	**N**	**Difference in mean MAP (mmHg) with each confounder (95% CI)**	***P*-value**
Highest household social class	368		
I		reference	
II		1.29 (-0.42, 2.99)	0.14
III (non-manual)		0.89 (-1.15, 2.93)	0.39
III (manual)		0.78 (-2.09, 3.65)	0.59
IV		0.44 (-5.08, 5.96)	0.87
Maternal education	377		
CSE		reference	
Vocational		-1.93 (-5.35, 1.49)	0.27
O-Level		-1.84 (-4.52, 0.84)	0.18
A-Level		-0.94 (-3.60, 1.71)	0.49
Degree		-2.68 (-5.42, 0.06)	0.06
Gender	415		
Male		reference	
Female		2.63 (1.30, 3.96)	0.0001
Age at 17 (years)	415	0.10 (-0.59, 0.78)	0.78
Smoking status	414		
Never		reference	
Ever		2.63 (1.30, 3.96)	0.0001
Weekly exercise	298		
Never/rarely/<2 per week		reference	
≥2 times per week		0.10 (-0.59, 0.78)	0.78

ALSPAC = Avon Longitudinal Study of Parents and Children; BMI = body mass index; CI = confidence interval; CSE = certificate of secondary education; MAP = mean arterial pressure
Estimates represent the difference in MAP (mmHg) per unit increase in each continuous confounder or level increase in each categorical or binary confounder

Supplementary Table S17. Associations between confounders and mean cIMT in ALSPAC 21-year RbG group

**Confounder**	**N**	**Difference in mean cIMT (mm) with each confounder (95% CI)**	***P*-value**
Highest household social class	369		
I		reference	
II		-0.005 (-0.02, 0.01)	0.38
III (non-manual)		0.01 (-0.01, 0.02)	0.31
III (manual)		-0.002 (-0.02, 0.02)	0.85
IV		-0.01 (-0.05, 0.02)	0.38
Maternal education			
CSE	379	reference	
Vocational		0.001 (-0.02, 0.02)	0.90
O-Level		-0.01 (-0.02, 0.01)	0.46
A-Level		-0.01 (-0.02, 0.01)	0.49
Degree		-0.01 (-0.03, 0.01)	0.34
Gender	417		
Male		reference	
Female		0.01 (0.001, 0.02)	0.04
Age at 17 (years)	417	0.0004 (-0.004, 0.005)	0.86
Smoking status	416		
Never		reference	
Ever		0.01 (-0.004, 0.02)	0.21
Weekly exercise	299		
Never/rarely/<2 per week		reference	
≥2 times per week		0.01 (-0.005, 0.02)	0.29

ALSPAC = Avon Longitudinal Study of Parents and Children; BMI = body mass index; CI = confidence interval; cIMT = carotid intima media thickness; CSE = certificate of secondary education

Estimates represent the difference in mean cIMT (mm) per unit increase in each continuous confounder or level increase in each categorical or binary confounder

Supplementary Table S18. Associations between confounders and carotid-femoral PWV in ALSPAC 21-year RbG group

**Confounder**	**N**	**Difference in PWV (%) with each confounder (95% CI)***	***P*-value**
Highest household social class	359		
I		reference	
II		1.19 (-2.10, 4.59)	0.48
III (non-manual)		4.64 (0.53, 8.92)	0.03
III (manual)		5.61 (-0.16, 11.72)	0.06
IV		15.28 (3.64, 28.23)	0.01
Maternal education	367		
CSE		reference	
Vocational		-2.62 (-9.06, 4.26)	0.44
O-Level		0.80 (-4.38, 6.26)	0.77
A-Level		-1.31 (-6.34, 3.99)	0.62
Degree		-3.71 (-8.74, 1.60)	0.17
Gender	404		
Male		reference	
Female		2.84 (0.16, 5.593)	0.04
Age at clinic (years)	404	0.44 (-0.91, 1.81)	0.52
Smoking status	403		
Never		reference	
Ever		-0.27 (-3.58, 3.16)	0.88
Weekly exercise	289		
Never/rarely/<2 per week		reference	
≥2 times per week		-2.73 (-5.50, 0.13)	0.06

ALSPAC = Avon Longitudinal Study of Parents and Children; BMI = body mass index; CI = confidence interval; CSE = certificate of secondary education; PWV = pulse wave velocity
**Percentage change in PWV (m/s) per unit increase in each continuous confounder or level increase in each categorical or binary confounder. For analyses, carotid-femoral PWV was log-transformed for normalization of the residuals; therefore, differences and confidence intervals were back-transformed and represent the mean percentage (%) difference in PWV.*

Supplementary Table S19. Associations between confounders and SVR in ALSPAC 21-year RbG group

**Confounder**	**N**	**Difference in mean SVR (mmHg/L/min.m$^{1.83}$) with each confounder (95% CI)***	***P*-value**
Highest household social class	340		
I		reference	
II		-0.09 (-1.99, 1.80)	0.92
III (non-manual)		-1.18 (-3.48, 1.13)	0.32
III (manual)		-1.30 (-4.68, 2.08)	0.45
IV		-4.45 (-10.43, 1.53)	0.14
Maternal education	349		
CSE		reference	
Vocational		1.01 (-2.85, 4.88)	0.61
O-Level		1.80 (-1.24, 4.85)	0.24
A-Level		1.37 (-1.65, 4.40)	0.37
Degree		1.75 (-1.35, 4.85)	0.27
Gender	386		
Male		reference	
Female		1.38 (-0.15, 2.91)	0.08
Age at clinic (years)	386	0.67 (-0.14, 1.48)	0.11
Smoking status	385		
Never		reference	
Ever		-0.06 (-2.03, 1.92)	0.95
Weekly exercise	285		
Never/rarely/<2 per week		reference	
≥2 times per week		1.97 (0.24, 3.70)	0.03

ALSPAC = Avon Longitudinal Study of Parents and Children; BMI = body mass index; CI = confidence interval; CSE = certificate of secondary education; SVR = systemic vascular resistance
Estimates represent the difference in SVR (mmHg/L/min.m$^{1.83}$) per unit increase in each continuous confounder or level increase in each categorical or binary confounder
**Adjusted for height by multiplying SVR by height$^{1.83}$*

Supplementary Table S20. Associations between confounders and TAC in ALSPAC 21-year RbG group

Confounder	N	Difference in mean TAC ($mL/mmHg/m^{1.83}$) with each confounder (95% CI)*	*P*-value
Highest household social class	340		
I		reference	
II		-0.01 (-0.03, 0.01)	0.20
III (non-manual)		-0.002 (-0.03, 0.02)	0.88
III (manual)		-0.02 (-0.06, 0.01)	0.25
IV		-0.02 (-0.08, 0.05)	0.62
Maternal education	349		
CSE		reference	
Vocational		-0.01 (-0.05, 0.03)	0.63
O-Level		-0.01 (-0.04, 0.02)	0.41
A-Level		-0.005 (-0.04, 0.03)	0.77
Degree		-0.01 (-0.05, 0.02)	0.40
Gender	386		
Male		reference	
Female		-0.01 (-0.03, 0.002)	0.08
Age at clinic (years)	386	0.01 (-0.004, 0.01)	0.26
Smoking status	385		
Never		reference	
Ever		0.02 (-0.01, 0.04)	0.14
Weekly exercise	285		
Never/rarely/<2 per week		reference	
≥2 times per week		0.01 (-0.01, 0.03)	0.22

ALSPAC = Avon Longitudinal Study of Parents and Children; BMI = body mass index; CI = confidence interval; CSE = certificate of secondary education; TAC = total arterial compliance
Estimates represent the difference in TAC ($mL/mmHg/m^{1.83}$) per unit increase in each continuous confounder or level increase in each categorical or binary confounder
Adjusted for height by dividing TAC measurements by $height^{1.83}$

Supplementary Table S21. Associations between confounders and LVMI in ALSPAC 21-year RbG group

**Confounder**	**N**	**Difference in mean LVMI (g/m$^{2.7}$) with each confounder (95% CI)**	***P*-value**
Highest household social class	340		
I		reference	
II		0.03 (-1.03, 1.10)	0.96
III (non-manual)		1.41 (0.11, 2.70)	0.03
III (manual)		0.08 (-1.82, 1.98)	0.93
IV		2.85 (-0.51, 6.21)	0.10
Maternal education	349		
CSE		reference	
Vocational		0.98 (-1.22, 3.18)	0.38
O-Level		-0.54 (-2.27, 1.19)	0.54
A-Level		-0.81 (-2.53, 0.91)	0.35
Degree		-1.09 (-2.86, 0.67)	0.22
Gender	386		
Male		reference	
Female		3.63 (2.83, 4.43)	1.89×10^{-17}
Age at clinic (years)	386	0.35 (-0.12, 0.81)	0.14
Smoking status	385		
Never		reference	
Ever		0.71 (-0.42, 1.83)	0.22
Weekly exercise	285		
Never/rarely/<2 per week		reference	
≥2 times per week		1.87 (0.91, 2.82)	0.0001

ALSPAC = Avon Longitudinal Study of Parents and Children; BMI = body mass index; CI = confidence interval; CSE = certificate of secondary education; LVMI = left ventricular mass indexed to height$^{2.7}$

Estimates represent the difference in LVMI (g/m$^{2.7}$) per unit increase in each continuous confounder or level increase in each categorical or binary confounder

Supplementary Table S22. Associations between confounders and heart rate in ALSPAC 21-year RbG group

**Confounder**	**N**	**Difference in mean heart rate (bpm) with each confounder (95% CI)**	***P*-value**
Highest household social class	361		
I		reference	
II		2.92 (0.49, 5.35)	0.02
III (non-manual)		2.43 (-0.50, 5.36)	0.10
III (manual)		5.58 (1.45, 9.71)	0.01
IV		2.17 (-5.11, 9.46)	0.56
Maternal education	369		
CSE		reference	
Vocational		-2.24 (-7.19, 2.71)	0.37
O-Level		1.39 (-2.46, 5.23)	0.48
A-Level		0.76 (-3.05, 4.56)	0.70
Degree		0.18 (-3.72, 4.09)	0.93
Gender	406		
Male		reference	
Female		-3.69 (-5.60, -1.78)	0.0002
Age at clinic (years)	406	-1.15 (-2.13, -0.16)	0.02
Smoking status	405		
Never		reference	
Ever		-0.36 (-2.82, 2.09)	0.77
Weekly exercise	290		
Never/rarely/<2 per week		reference	
≥2 times per week		-5.96 (-8.06, -3.86)	$5.36x10^{-08}$

ALSPAC = Avon Longitudinal Study of Parents and Children; BMI = body mass index; CI = confidence interval; CSE = certificate of secondary education
Estimates represent the difference in heart rate (bpm) per unit increase in each continuous confounder or level increase in each categorical or binary confounder

Supplementary Table S23. Associations between confounders and SV in ALSPAC 21-year RbG group

**Confounder**	**N**	**Difference in mean SV (ml/m$^{2.04}$) with each confounder (95% CI)**	***P*-value**
Highest household social class	340		
I		reference	
II		-0.74 (-1.88, 0.39)	0.20
III (non-manual)		0.30 (-1.08, 1.68)	0.67
III (manual)		-0.48 (-2.50, 1.55)	0.64
IV		1.44 (-2.13, 5.02)	0.43
Maternal education	349		
CSE		reference	
Vocational		0.98 (-1.33, 3.30)	0.40
O-Level		-1.14 (-2.97, 0.68)	0.22
A-Level		-0.95 (-2.76, 0.86)	0.30
Degree		-1.14 (-3.00, 0.72)	0.23
Gender	386		
Male		reference	
Female		1.07 (0.16, 1.99)	0.02
Age at clinic (years)	386	0.39 (-0.10, 0.87)	0.12
Smoking status	385		
Never		reference	
Ever		0.91 (-0.27, 2.09)	0.13
Weekly exercise	298		
Never/rarely/<2 per week		reference	
≥2 times per week		1.54 (0.50, 2.59)	0.004

ALSPAC = Avon Longitudinal Study of Parents and Children; BMI = body mass index; CI = confidence interval; CSE = certificate of secondary education; SV = stroke volume
Estimates represent the difference in SV (ml/m$^{2.04}$) per unit increase in each continuous confounder or level increase in each categorical or binary confounder

Supplementary Table S24. Associations between confounders and CO in ALSPAC 21-year RbG group

Confounder	N	Difference in mean CO (l/min/m$^{1.83}$) with each confounder (95% CI)	*P*-value
Highest household social class	340		
I		reference	
II		0.04 (-0.07, 0.14)	0.48
III (non-manual)		0.11 (-0.01, 0.24)	0.07
III (manual)		0.04 (-0.14, 0.23)	0.63
IV		0.38 (0.06, 0.70)	0.02
Maternal education	349		
CSE		reference	
Vocational		-0.01 (-0.22, 0.19)	0.89
O-Level		-0.08 (-0.24, 0.08)	0.34
A-Level		-0.09 (-0.26, 0.07)	0.26
Degree		-0.12 (-0.28, 0.05)	0.17
Gender	386		
Male		reference	
Female		-0.004 (-0.09, 0.08)	0.92
Age at clinic (years)	386	-0.02 (-0.06, 0.02)	0.39
Smoking status	385		
Never		reference	
Ever		-0.02 (-0.13, 0.09)	0.75
Weekly exercise	285		
Never/rarely/<2 per week		reference	
≥2 times per week		-0.12 (-0.21, -0.02)	0.01

ALSPAC = Avon Longitudinal Study of Parents and Children; BMI = body mass index; CI = confidence interval; CSE = certificate of secondary education; CO = cardiac output
Estimates represent the difference in CO (l/min/m$^{1.83}$) per unit increase in each continuous confounder or level increase in each categorical or binary confounder

Supplementary Table S25. Associations between RbG groups and confounders in ALSPAC 21-year RbG group

**Confounder**	**N**	**OR (95% CI)**	***P*-value**
Highest household social class	370		
I		1.00 (reference)	
II		0.91 (0.55, 1.52)	0.72
III (non-manual)		1.65 (0.88, 3.09)	0.12
III (manual)		1.46 (0.60, 3.52)	0.40
IV		0.64 (0.14, 3.04)	0.58
Maternal education	380		
CSE		1.00 (reference)	
Vocational		1.01 (0.34, 3.00)	0.99
O-Level		0.56 (0.24, 1.30)	0.18
A-Level		0.64 (0.28, 1.49)	0.30
Degree		0.42 (0.18, 0.98)	0.05
Gender	418		
Male		1.00 (reference)	
Female		1.21 (0.80, 1.81)	0.37
Age	418	0.97 (0.79, 1.19)	0.76
Smoking status	417		
Never		1.00 (reference)	
Ever		1.07 (0.64, 1.78)	0.80
Weekly exercise	299		
Never/rarely/<2 per week		1.00 (reference)	
≥2 times per week		1.33 (0.83, 2.12)	0.23

ALSPAC = Avon Longitudinal Study of Parents and Children; BMI = body mass index; CI = confidence interval; CSE = certificate of secondary education; RbG = recall-by-genotype
Estimates represent the odds of being in the upper of the two RbG groups per unit increase in each continuous confounder or level increase in each categorical or binary confounder

Supplementary Table S26. Impact of BP and heart rate in the associations of BMI with mean cIMT, PWV and LVMI in ALSPAC using MR and RbG analyses

**Outcome (units)**	**N**	**Difference in mean outcome (95% CI)**	***P*-value**
MR analysis			
Mean cIMT (mm)	3059	0.001 (-0.001, 0.003)	0.37
Carotid-femoral PWV (%)*	2491	-0.45 (-1.10, 0.21)	0.18
LVMI ($g/m^{2.7}$)	1386	0.90 (0.45, 1.35)	0.0001
RbG analysis			
Mean cIMT (mm)	404	-0.003 (-0.01, 0.01)	0.51
Carotid-femoral PWV (%)*	404	1.56 (-0.71, 3.89)	0.18
LVMI ($g/m^{2.7}$)	376	0.95 (0.23, 1.67)	0.01

ALSPAC = Avon Longitudinal Study of Parents and Children; BMI = body mass index; CI = confidence interval; LVMI = left ventricular mass indexed to height$^{2.7}$; PWV = pulse wave velocity; RbG = recall-by-genotype

Estimates represent difference in each cardiovascular outcome per unit (kg/m^2) higher BMI (with MR analysis) or per 3.58kg/m^2 higher BMI (in RbG analysis), adjusted for SBP and heart rate

**Percentage change in PWV (m/s) per unit (kg/m^2) higher BMI (with MR analysis) or per 3.58kg/m^2 higher BMI (in RbG analysis), adjusted for SBP and heart rate. For analyses, carotid-femoral PWV was log-transformed for normalization of the residuals; therefore, differences and confidence intervals were back-transformed and represent the mean percentage (%) difference in PWV.*

Supplementary Table S27. Association of weighted GRS (comprising 97 SNPs) with height, height-squared and BMI (adjusted for height and height-squared) in ALSPAC 17-year clinic

**Outcome (units)**	**N**	**Difference in mean outcome per unit increase in the GRS (95% CI)**	***P*-value**	**R^2**
Height (m)	3406	-0.005 (-0.06, 0.05)	0.86	$9.65x10^{-06}$
Height-squared (m^2)	3406	-2.00 (-19.47, 15.48)	0.82	$1.47x10^{-05}$
BMI (kg/m^2)*	3404	0.12 (0.10, 0.14)	$8.49x10^{-28}$	0.04

ALSPAC = Avon Longitudinal Study of Parents and Children; BMI = body mass index; CI = confidence interval; GRS = genetic risk score; SNP = single nucleotide polymorphism
**Adjusted for height and height-squared*

Supplementary Table S28. Multivariable regression associations between BMI and cardiovascular phenotypes in ALSPAC 17-year clinic adjusting for height and height-squared

**Outcome (units)**	**N**	**Difference in mean outcome per 1kg/m^2 higher BMI (95% CI)***	***P*-value**	**N**	**Difference in mean outcome per 1kg/m^2 higher BMI (95% CI)†**	***P*-value**	**N**	**Difference in mean outcome per 1kg/m^2 higher BMI (95% CI)‡**	***P*-value**
SBP (mmHg)	3108	0.91 (0.83, 0.99)	1.93×10^{-102}	2389	0.87 (0.77, 0.97)	2.79×10^{-67}	1033	0.87 (0.72, 1.02)	1.20×10^{-27}
DBP (mmHg)	3108	0.52 (0.46, 0.57)	9.08×10^{-76}	2389	0.49 (0.42, 0.56)	7.28×10^{-44}	1033	0.48 (0.37, 0.59)	8.25×10^{-18}
PP (mmHg)	3108	0.39 (0.32, 0.47)	2.28×10^{-23}	2389	0.38 (0.29, 0.46)	8.85×10^{-17}	1033	0.39 (0.25, 0.53)	4.81×10^{-08}
MAP (mmHg)	3108	0.65 (0.59, 0.70)	1.15×10^{-118}	2389	0.62 (0.55, 0.69)	2.06×10^{-69}	1033	0.61 (0.50, 0.71)	4.20×10^{-28}
Mean cIMT (mm)	3079	0.0002 (-0.0002, 0.001)	0.44	2368	0.00003 (-0.0005, 0.001)	0.92	1028	0.0004 (-0.0003, 0.001)	0.26
Carotid-femoral PWV (%)§	2495	-0.004 (-0.12, 0.12)	0.95	1957	-0.03 (-0.17, 0.12)	0.72	867	-0.16 (-0.38, 0.06)	0.16
LVMI (g/m$^{2.7}$)	1420	0.80 (0.73, 0.87)	2.03×10^{-108}	1151	0.79 (0.71, 0.87)	3.00×10^{-83}	569	0.91 (0.79, 1.03)	7.65×10^{-53}
Heart rate (bpm)	3108	0.18 (0.10, 0.27)	2.89×10^{-05}	2389	0.19 (0.08, 0.30)	0.0004	1033	0.22 (0.06, 0.38)	5.91×10^{-03}

ALSPAC = Avon Longitudinal Study of Parents and Children; BMI = body mass index; CI = confidence interval; cIMT = carotid intima-media thickness; DBP = diastolic blood pressure; LVMI = left ventricular mass indexed to height$^{2.7}$; PWV = pulse wave velocity; SBP = systolic blood pressure

**Unadjusted*

†Adjusted for age, gender, smoking and dietary intake of the participant and maternal education and highest household social class

‡Additionally adjusted for physical activity of the participant

§Percentage change in PWV (m/s) per 1kg/m^2 higher in BMI. For analyses, carotid-femoral PWV was log-transformed for normalization of the residuals; therefore, differences and confidence intervals were back-transformed and represent the mean percentage (%) difference in PWV.

Supplementary Table S29. Mendelian randomization analyses of the association between BMI and cardiovascular phenotypes in ALSPAC 17-year clinic (using 97 SNPs) with height and height-squared adjustments

**Outcome (units)**	**N**	**Difference in mean outcome per 1kg/m² higher BMI (95% CI)**	***P*-value**	***F*-stat**	***P*-value for difference in multivariable regression and MR analyses***
SBP (mmHg)	3108	0.83 (0.39, 1.26)	0.0002	115.80	0.70
DBP (mmHg)	3108	0.29 (0.001, 0.59)	0.05	115.80	0.12
PP (mmHg)	3108	0.53 (0.13, 0.94)	0.01	115.80	0.49
MAP (mmHg)	3108	0.47 (0.18, 0.76)	0.001	115.80	0.22
Mean cIMT (mm)	3079	0.002 (-0.005, 0.004)	0.13	122.85	0.16
Carotid-femoral PWV (%)†	2495	-0.04 (-0.70, 0.62)	0.90	86.47	0.91
LVMI ($g/m^{2.7}$)	1420	1.06 (0.61, 1.51)	3.94×10^{-06}	36.86	0.23
Heart rate (bpm)	3108	-0.05 (-0.50, 0.40)	0.82	115.80	0.30

ALSPAC = Avon Longitudinal Study of Parents and Children; BMI = body mass index; CI = confidence interval; cIMT = carotid intima-media thickness; DBP = diastolic blood pressure; LVMI = left ventricular mass indexed to height$^{2.7}$; MR = Mendelian randomization; PWV = pulse wave velocity; SBP = systolic blood pressure; SNP = single nucleotide polymorphism

**P-value obtained from Durbin-Wu-Hausman test of heterogeneity between estimates obtained from unadjusted multivariable regression and MR analyses*

†Percentage change in PWV (m/s) per 1kg/m² higher in BMI. For analyses, carotid-femoral PWV was log-transformed for normalization of the residuals; therefore, differences and confidence intervals were back-transformed and represent the mean percentage (%) difference in PWV.

Supplementary Table S30. Mendelian randomization analyses of the association between BMI and cardiovascular phenotypes in ALSPAC 17-year clinic using MR-Egger

**Outcome (units)**	**Estimate of pleiotropy (95% CI)***	***P***	**IVW (95% CI)†**	***P***	**MR-Egger (95% CI)‡**	***P***	**Weighted median (95% CI)§**	***P***
SBP (mmHg)	0.02 (-0.14, 0.19)	0.79	0.83 (0.26, 1.40)	0.005	0.66 (-0.73, 2.06)	0.35	-0.12 (-0.52, 1.24)	0.45
DBP (mmHg)	-0.01 (-0.10, 0.09)	0.91	0.32 (-0.002, 0.64)	0.05	0.36 (-0.42, 1.14)	0.36	0.27 (-0.27, 0.88)	0.33
PP (mmHg)	0.03 (-0.13, 0.18)	0.72	0.52 (-0.01, 1.04)	0.06	0.30 (-0.99, 1.59)	0.64	0.18 (-0.67, 0.99)	0.67
MAP (mmHg)	0.004 (-0.09, 0.10)	0.94	0.49 (0.15, 0.83)	0.005	0.43 (-0.42, 1.17)	0.27	0.14 (-0.26, 0.88)	0.30
Mean cIMT (mm)	0.0001 (-0.001, 0.001)	0.69	0.002 (-0.001, 0.004)	0.14	0.001 (-0.005, 0.01)	0.81	0.001 (-0.002, 0.01)	0.42
Carotid-femoral PWV (%)‖	-0.001 (-0.20, 0.20)	0.99	-0.10 (-1.00, 1.01)	0.81	-0.10 (-1.98, 2.02)	0.93	-1.00 (-1.00, 1.01)	0.53
LVMI	0.01 (-0.13, 0.14)	0.94	0.93 (0.48, 1.37)	$8.62x10^{-05}$	0.89 (-0.21, 1.98)	0.11	0.89 (0.18, 1.64)	0.02
Heart rate (bpm)	0.08 (-0.08, 0.22)	0.24	-0.09 (-0.57, 0.39)	0.72	-0.73 (-1.89, 0.44)	0.22	-0.16 (-0.97, 0.64)	0.73

ALSPAC = Avon Longitudinal Study of Parents and Children; BMI = body mass index; CI = confidence interval; cIMT = carotid intima-media thickness; DBP = diastolic blood pressure; GRS = genetic risk score; IVW = inverse-variance weighted; LVMI = left ventricular mass indexed to height$^{2.7}$; PWV = pulse wave velocity; SBP = systolic blood pressure; SNP = single nucleotide polymorphism

**Estimated effect of directional pleiotropy, where the null hypothesis (estimate=0) suggests no directional pleiotropy, as predicted by the intercept term of MR-Egger regression*

Estimates represent the difference in each cardiovascular measure per unit (kg/m^2) increase in BMI, as predicted by the weighted GRS (comprising 97 SNPs) using:

†The inverse-variance weighted (IVW) method

‡MR-Egger regression, taking into account violations in the MR assumption of directional pleiotropy

§The weighted median approach, providing a causal estimate assuming that at most 50% of the SNPs included are invalid

‖Percentage change in PWV (m/s) per 1kg/m^2 higher in BMI. For analyses, carotid-femoral PWV was log-transformed for normalization of the residuals; therefore, differences and confidence intervals were back-transformed and represent the mean percentage (%) difference in PWV.

Supplementary Table S31. Association between BMI GRS (comprising 77 or 71 SNPs) or genome-wide GRS and BMI in ALSPAC 17-year clinic

**Number of SNPs included within the GRS or genome-wide GRS**	**N**	**Difference in mean BMI (kg/m²) per unit increase in the GRS (95% CI)**	***P*-value**	**R^2**
77 SNPs	3404	0.13 (0.10, 0.15)	4.65×10^{-24}	0.03
71 SNPs	3404	0.12 (0.10, 0.15)	3.21×10^{-21}	0.03
Genome-wide	3404	0.12 (0.10, 0.14)	5.31×10^{-35}	0.04

ALSPAC = Avon Longitudinal Study of Parents and Children; BMI = body mass index; CI = confidence interval; GRS = genetic risk score; SNP = single nucleotide polymorphism

Supplementary Table S32. Mendelian randomization analyses of the association between BMI and cardiovascular phenotypes in ALSPAC 17-year clinic (using 77 SNPs)

**Outcome (units)**	**N**	**Difference in mean outcome per 1kg/m^2 higher BMI (95% CI)**	***P*-value**	***F*-stat**	***P*-value for difference in multivariable regression and MR analyses***
SBP (mmHg)	3108	0.79 (0.28, 1.31)	0.003	103.01	0.81
DBP (mmHg)	3108	0.36 (0.05, 0.67)	0.02	103.01	0.29
PP (mmHg)	3108	0.44 (-0.06, 0.93)	0.08	103.01	0.68
MAP (mmHg)	3108	0.50 (0.19, 0.81)	0.002	103.01	0.40
Mean cIMT (mm)	3079	0.001 (-0.001, 0.003)	0.33	112.65	0.35
Carotid-femoral PWV (%)†	2495	-0.07 (-0.80, 0.66)	0.84	80.07	0.97
LVMI (g/m$^{2.7}$)	1420	1.10 (0.63, 1.57)	4.73×10^{-06}	34.04	0.18
Heart rate (bpm)	3108	-0.16 (-0.65, 0.33)	0.53	103.01	0.13

ALSPAC = Avon Longitudinal Study of Parents and Children; BMI = body mass index; CI = confidence interval; cIMT = carotid intima-media thickness; DBP = diastolic blood pressure; LVMI = left ventricular mass indexed to height$^{2.7}$; MR = Mendelian randomization; PWV = pulse wave velocity; SBP = systolic blood pressure; SNP = single nucleotide polymorphism

**P-value obtained from Durbin-Wu-Hausman test of heterogeneity between estimates obtained from unadjusted multivariable regression and MR analyses*

†Percentage change in PWV (m/s) per 1kg/m^2 higher in BMI. For analyses, carotid-femoral PWV was log-transformed for normalization of the residuals; therefore, differences and confidence intervals were back-transformed and represent the mean percentage (%) difference in PWV.

Supplementary Table S33. Mendelian randomization analyses of the association between BMI and cardiovascular phenotypes in ALSPAC 17-year clinic (using 71 SNPs)

**Outcome (units)**	**N**	**Difference in mean outcome per 1kg/m² higher BMI (95% CI)**	***P*-value**	***F*-stat**	***P*-value for difference in multivariable regression and MR analyses***
SBP (mmHg)	3108	0.86 (0.31, 1.41)	0.002	90.55	0.98
DBP (mmHg)	3108	0.37 (0.05, 0.70)	0.03	90.55	0.37
PP (mmHg)	3108	0.49 (-0.04, 1.02)	0.07	90.55	0.56
MAP (mmHg)	3108	0.54 (0.21, 0.87)	0.002	90.55	0.57
Mean cIMT (mm)	3079	0.001 (-0.001, 0.004)	0.21	98.13	0.23
Carotid-femoral PWV (%)†	2495	-0.06 (-0.81, 0.69)	0.87	76.22	0.96
LVMI ($g/m^{2.7}$)	1420	1.18 (0.69, 1.68)	$2.85x10^{-06}$	31.66	0.10
Heart rate (bpm)	3108	-0.04 (-0.56, 0.48)	0.88	90.55	0.34

ALSPAC = Avon Longitudinal Study of Parents and Children; BMI = body mass index; CI = confidence interval; cIMT = carotid intima-media thickness; DBP = diastolic blood pressure; LVMI = left ventricular mass indexed to height$^{2.7}$; MR = Mendelian randomization; PWV = pulse wave velocity; SBP = systolic blood pressure; SNP = single nucleotide polymorphism

**P-value obtained from Durbin-Wu-Hausman test of heterogeneity between estimates obtained from unadjusted multivariable regression and MR analyses*

†Percentage change in PWV (m/s) per 1kg/m² higher in BMI. For analyses, carotid-femoral PWV was log-transformed for normalization of the residuals; therefore, differences and confidence intervals were back-transformed and represent the mean percentage (%) difference in PWV.

Supplementary Table S34. Mendelian randomization analyses of the association between BMI and cardiovascular phenotypes in ALSPAC 17-year clinic (using the genome-wide GRS)

**Outcome (units)**	**N**	**Difference in mean outcome per 1kg/m^2 higher BMI (95% CI)**	***P*-value**	***F*-stat**	***P*-value for difference in multivariable regression and MR analyses***
SBP (mmHg)	3108	0.98 (0.54, 1.42)	1.16x10^{-05}	144.05	0.56
DBP (mmHg)	3108	0.28 (0.01, 0.54)	0.04	144.05	0.06
PP (mmHg)	3108	0.70 (0.28, 1.13)	0.001	144.05	0.08
MAP (mmHg)	3108	0.51 (0.25, 0.78)	0.0002	144.05	0.36
Mean cIMT (mm)	3079	0.001 (-0.001, 0.003)	0.25	142.40	0.26
Carotid-femoral PWV (%)†	2495	-0.23 (-0.86, 0.40)	0.47	108.54	0.64
LVMI (g/m$^{2.7}$)	1420	0.82 (0.50, 1.15)	6.99x10^{-07}	69.12	0.84
Heart rate (bpm)	3108	-0.30 (-0.72, 0.12)	0.16	144.05	0.01

ALSPAC = Avon Longitudinal Study of Parents and Children; BMI = body mass index; CI = confidence interval; cIMT = carotid intima-media thickness; DBP = diastolic blood pressure; LVMI = left ventricular mass indexed to height$^{2.7}$; MR = Mendelian randomization; PWV = pulse wave velocity; SBP = systolic blood pressure; SNP = single nucleotide polymorphism

**P-value obtained from Durbin-Wu-Hausman test of heterogeneity between estimates obtained from unadjusted multivariable regression and MR analyses*

†Percentage change in PWV (m/s) per 1kg/m^2 higher in BMI. For analyses, carotid-femoral PWV was log-transformed for normalization of the residuals; therefore, differences and confidence intervals were back-transformed and represent the mean percentage (%) difference in PWV.

SUPPLEMENTARY FIGURES

Supplementary Figure S1. Association between weighted GRS (comprising 97 SNPs) and BMI in ALSPAC 17-year clinic

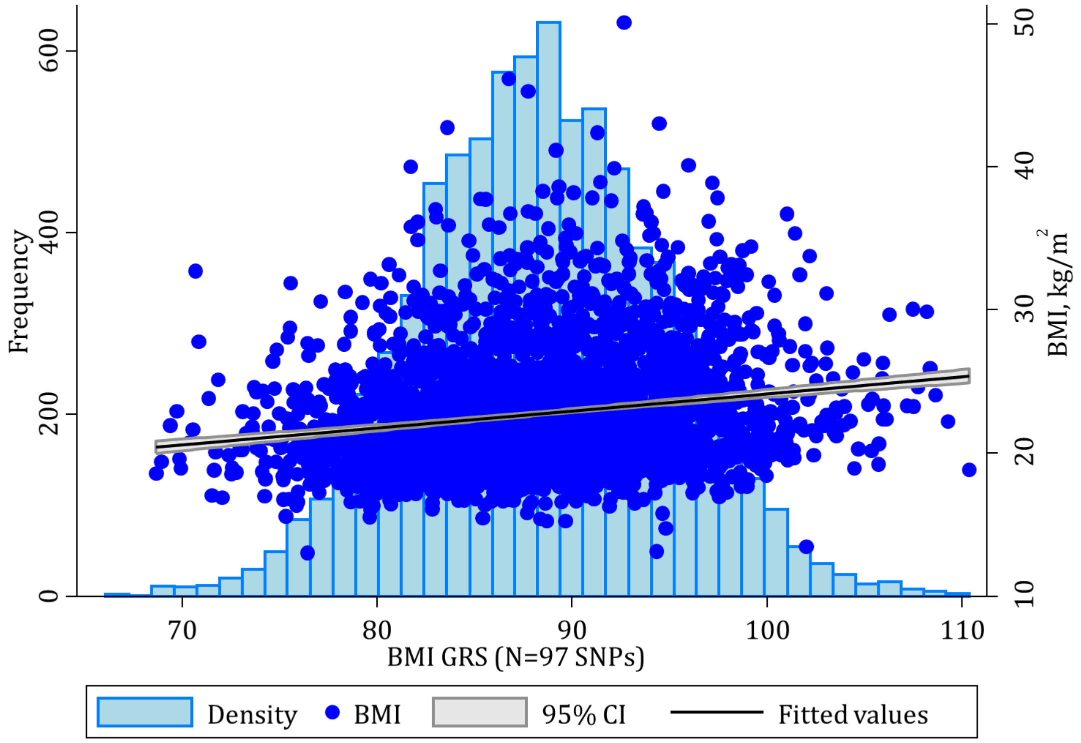

The light blue histogram represents the weighted GRS (comprising 97 SNPs) distribution (frequency, left-hand axis). The dark blue scatter plot and linear trend with corresponding 95% confidence intervals represent the association between the same weighted GRS and BMI (kg/m^2, right-hand axis).

Supplementary Figure S2. Association between RbG groups and BMI in ALSPAC 21-year RbG group

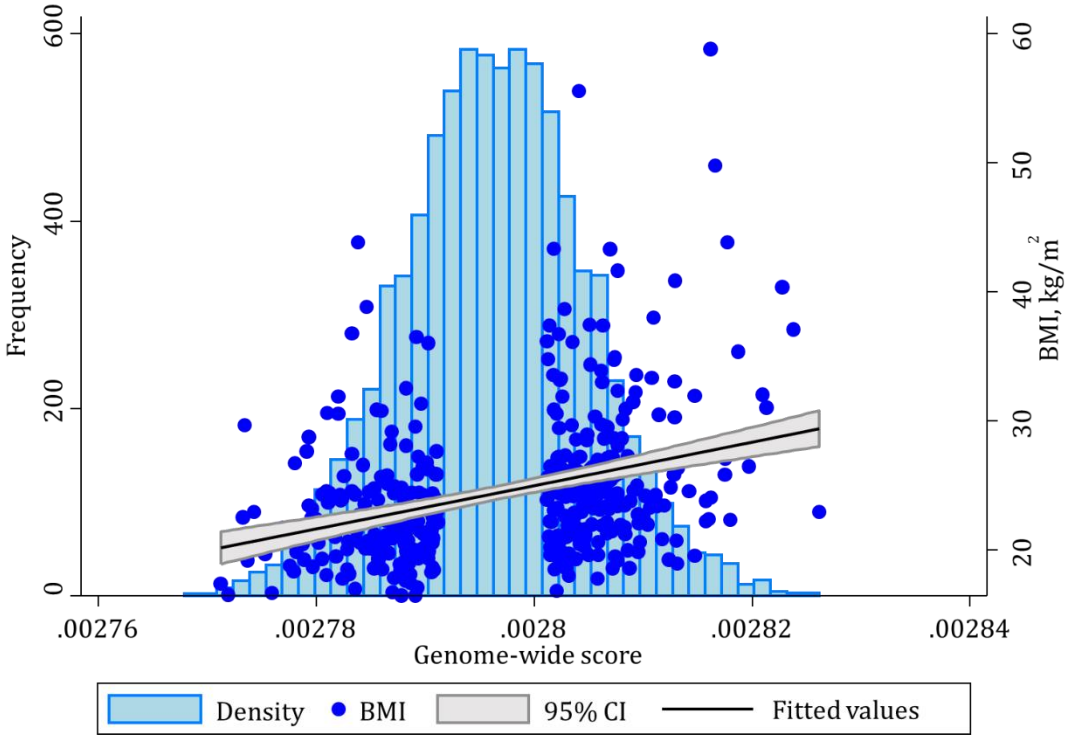

The light blue histogram represents the genome-wide GRS distribution (frequency, left-hand axis). The dark blue scatter plot and linear trend with corresponding 95% confidence intervals represent the association between the same genome-wide GRS and BMI (kg/m², right-hand axis) included in the RbG groups.

Supplementary Figure S3. Funnel and forest plot of IVW and MR-Egger results for the association between BMI and SBP in ALSPAC 17-year clinic

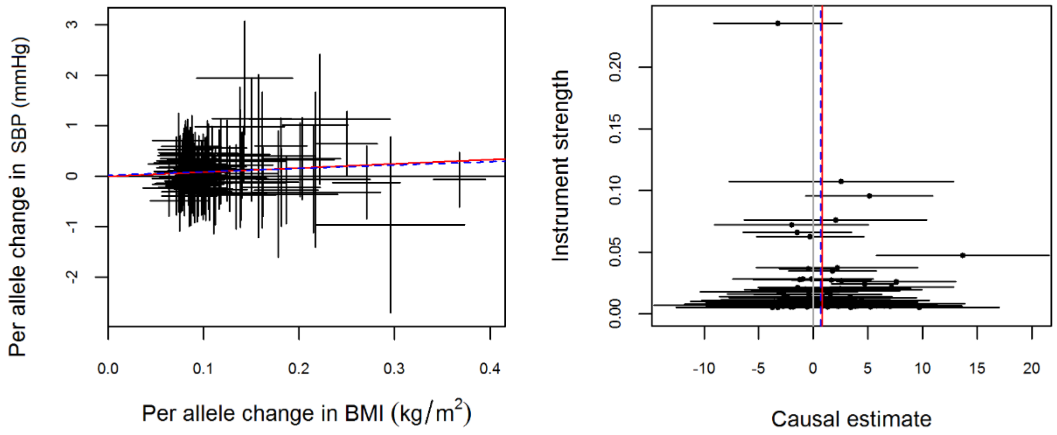

Supplementary Figure S4. Funnel and forest plot of IVW and MR-Egger results for the association between BMI and DBP in ALSPAC 17-year clinic

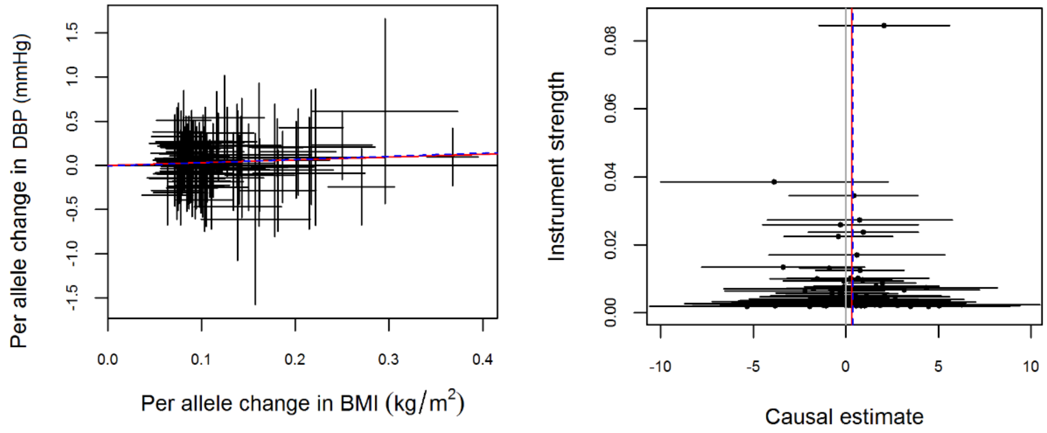

Supplementary Figure S5. Funnel and forest plot of IVW and MR-Egger results for the association between BMI and PP in ALSPAC 17-year clinic

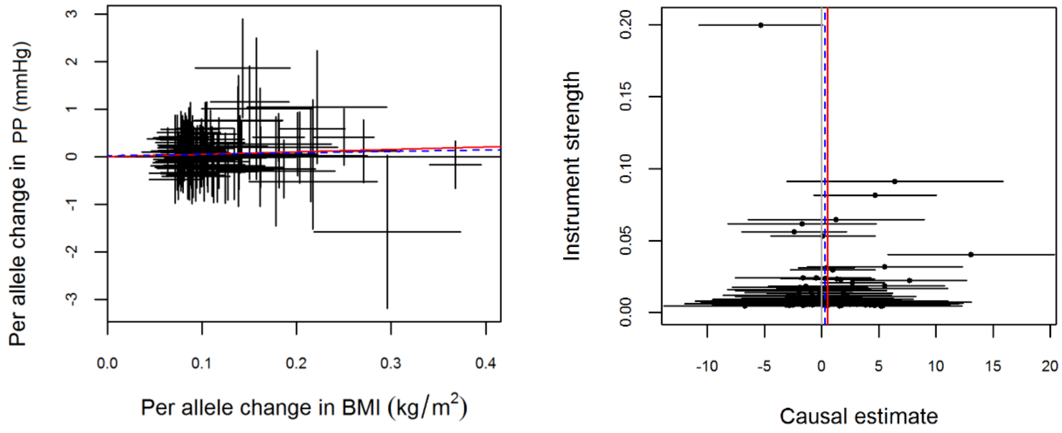

Supplementary Figure S6. Funnel and forest plot of IVW and MR-Egger results for the association between BMI and MAP in ALSPAC 17-year clinic

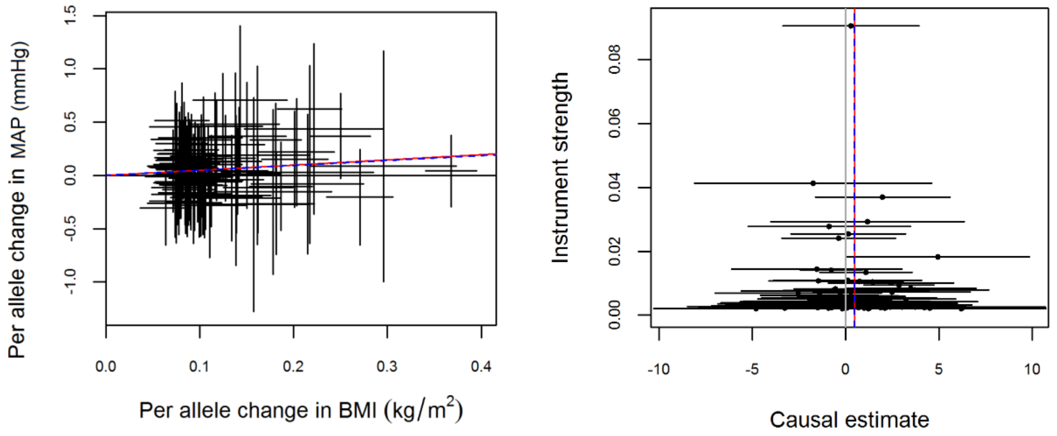

Supplementary Figure S7. Funnel and forest plot of IVW and MR-Egger results for the association between BMI and LVMI in ALSPAC 17-year clinic

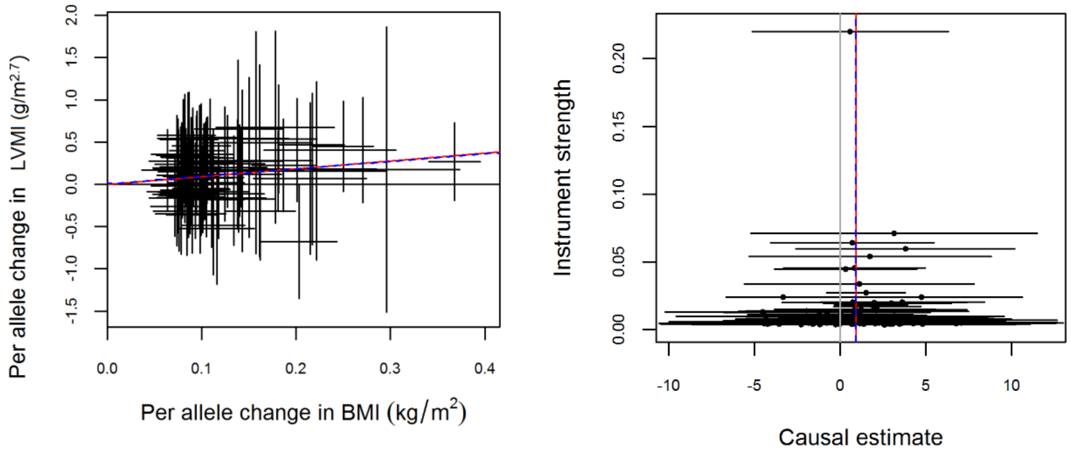

Supplementary Figure S8. Association between BMI GRS (comprising 77 SNPs) and BMI in ALSPAC 17-year clinic

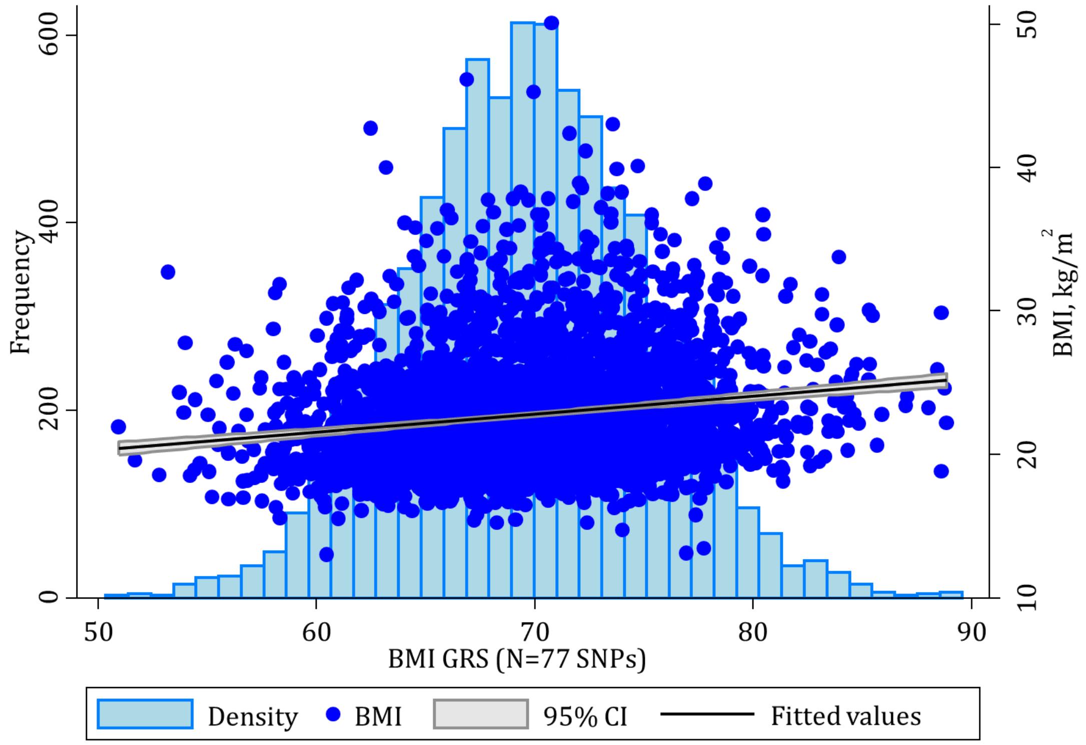

The light blue histogram represents the BMI GRS (comprising 77 SNPs) distribution (frequency, left-hand axis). The mid-blue scatter plot and linear trend with corresponding 95% confidence intervals represent the association between the same BMI GRS and BMI (kg/m², right-hand axis).

Supplementary Figure S9. Association between BMI GRS (comprising 71 SNPs) and BMI in ALSPAC 17-year clinic

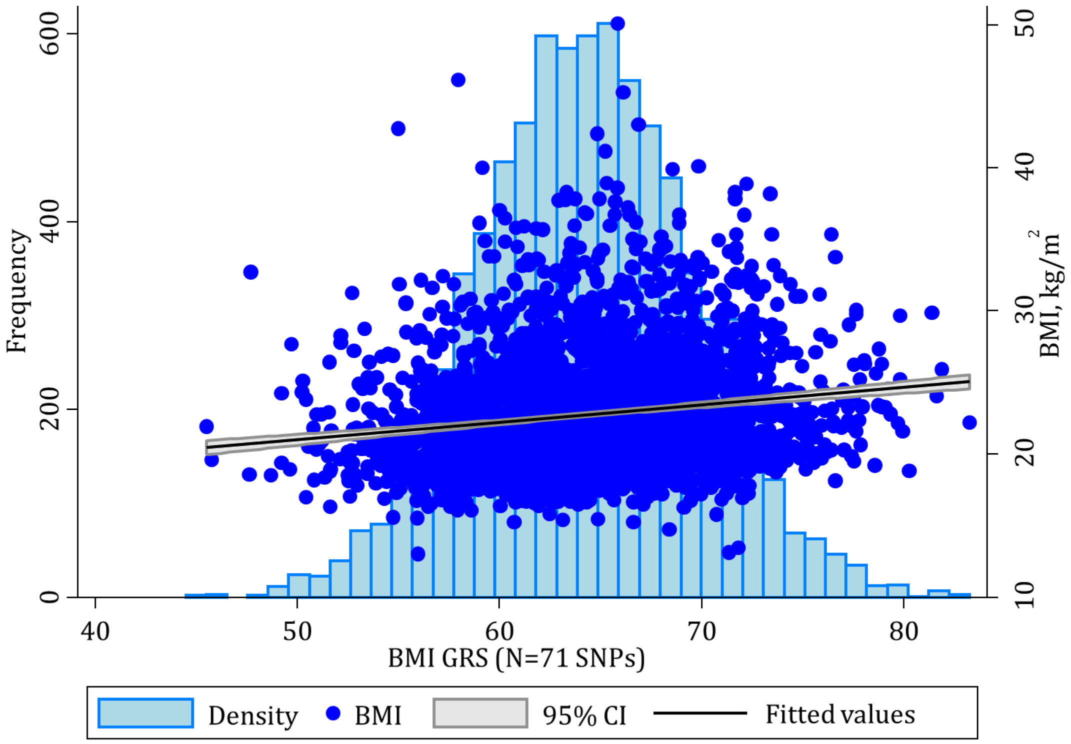

The light blue histogram represents the BMI GRS (comprising 71 SNPs) distribution (frequency, left-hand axis). The mid-blue scatter plot and linear trend with corresponding 95% confidence intervals represent the association between the same BMI GRS and BMI (kg/m^2, right-hand axis).

Supplementary Figure S10. Association between BMI genome-wide GRS and BMI in ALSPAC 17-year clinic

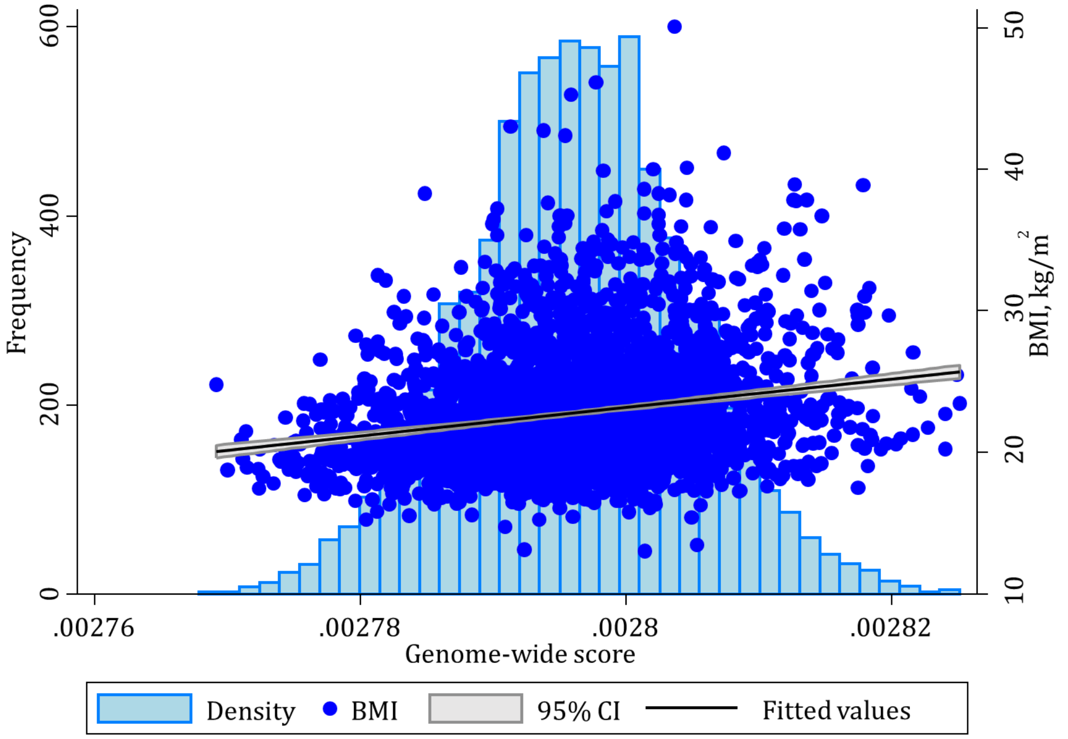

The light blue histogram represents the BMI genome-wide GRS distribution (frequency, left-hand axis). The mid-blue scatter plot and linear trend with corresponding 95% confidence intervals represent the association between the same BMI genome-wide GRS and BMI (kg/m², right-hand axis).

SUPPLEMENTARY REFERENCES

1. O'Brien E, Asmar R, Beilin L, Imai Y, Mallion JM, Mancia G, Mengden T, Myers M, Padfield P, Palatini P, Parati G, Pickering T, Redon J, Staessen J, Stergiou G, Verdecchia P; European Society of Hypertension Working Group on Blood Pressure Monitoring. European Society of Hypertension recommendations for conventional, ambulatory and home blood pressure measurement. *Journal of Hypertension* 2003;21(5):821-48.
2. Lang RM, Bierig M, Devereux RB, Flachskampf FA, Foster E, Pellikka PA, Picard MH, Roman MJ, Seward J, Shanewise JS, Solomon SD, Spencer KT, Sutton MS, Stewart WJ. Recommendations for chamber quantification: A report from the american society of echocardiography's guidelines and standards committee and the chamber quantification writing group, developed in conjunction with the european association of echocardiography, a branch of the european society of cardiology. *J Am Soc Echocardiogr*. 2005;18:1440-1463.
3. Steeden JA, Atkinson D, Hansen MS, Taylor AM, Muthurangu V.. Rapid Flow Assessment of Congenital Heart Disease with High-Spatiotemporal-Resolution Gated Spiral Phase-Contrast MR Imaging. *Radiology* 2011;260(1):79-87. doi: 10.1148/radiol.11101844
4. Stergiopulos N, Meister JJ, Westerhof N. Simple and accurate way for estimating total and segmental arterial compliance: the pulse pressure method. *Ann Biomed Eng* 1994;22:392-97.
5. Paternoster L, Howe LD, Tilling K, Weedon MN, Freathy RM, Frayling TM, Kemp JP, Davey Smith G, Timpson NJ, Ring SM, Evans DM, Lawlor DA. Adult height variants affect birth length and growth rate in children. *Hum Mol Genet*. 2011;20:4069-4075.
6. Office of Population Censuses and Surveys. *Standard Occupational Classification Volume 3*. London: HMSO; 1991.
7. Mattocks C, Ness A, Leary S, Tilling K, Blair SN, Shield J, Deere K, Saunders J, Kirkby J, Smith GD, Wells J, Wareham N, Reilly J, Riddoch C. Use of accelerometers in a large field-based study of children: protocols, design issues, and effects on precision. *J Phys Act Health*. 2008;5:S98-S111.
8. Anderson EL, Tilling K, Fraser A, Macdonald-Wallis C, Emmett P, Cribb V, Northstone K, Lawlor DA, Howe LD. Estimating trajectories of energy intake through childhood and adolescence using linear-spline multilevel models. *Epidemiology*. 2013;24:507-515.
9. Speliotes EK, Willer CJ, Berndt SI, Monda KL, Thorleifsson G, Jackson AU, Allen HL, Lindgren CM, Luan Ja, Magi R, Randall JC, Vedantam S, Winkler TW, Qi L, Workalemahu T, Heid IM, Steinthorsdottir V, Stringham HM, Weedon MN, Wheeler E, Wood AR, Ferreira T, Weyant RJ, Segre AV, Estrada K, Liang L, Nemesh J, Park J-H, Gustafsson S, Kilpelainen TO, Yang J, Bouatia-Naji N, Esko T, Feitosa MF, Kutalik Z, Mangino M, Raychaudhuri S, Scherag A, Smith AV, Welch R, Zhao JH, Aben KK, Absher DM, Amin N, Dixon AL, Fisher E, Glazer NL, Goddard ME, Heard-Costa NL, Hoesel V, Hottenga J-J, Johansson A, Johnson T, Ketkar S, Lamina C, Li S, Moffatt MF, Myers RH, Narisu N, Perry JRB, Peters MJ, Preuss M, Ripatti S, Rivadeneira F, Sandholt C, Scott LJ, Timpson NJ, Tyrer JP, van Wingerden S, Watanabe RM, White CC, Wiklund F, Barlassina C, Chasman DI, Cooper MN, Jansson J-O, Lawrence RW, Pellikka N, Prokopenko I, Shi J, Thiering E, Alavere H, Alibrandi MTS, Almgren P, Arnold AM, Aspelund T, Atwood LD, Balkau B, Balmforth AJ, Bennett AJ, Ben-Shlomo Y, Bergman RN, Bergmann S, Biebermann H, Blakemore AIF, Boes T, Bonnycastle LL, Bornstein SR, Brown MJ, Buchanan TA, Busonero F, Campbell H, Cappuccio FP, Cavalcanti-Proenca C, Chen Y-DI, Chen C-M, Chines PS, Clarke R, Coin L, Connell J, Day INM, Heijer Md, Duan J, Ebrahim S, Elliott P, Elosua R, Eiriksdottir G, Erdos MR, Eriksson JG, Facheris MF, Felix SB, Fischer-Posovszky P, Folsom AR, Friedrich N, Freimer NB, Fu M, Gaget S, Gejman PV, Geus EJC, Gieger C, Gjesing AP, Goel A, Goyette P, Grallert H, Graszler J, Greenawalt DM, Groves CJ, Gudnason V, Guiducci C, Hartikainen A-L, Hassanali N, Hall AS, Havulinna AS, Hayward C, Heath AC, Hengstenberg C, Hicks AA, Hinney A, Hofman A, Homuth G, Hui J, Igl W, Iribarren C, Isomaa B, Jacobs KB, Jarick I, Jewell E, John U, Jorgensen T, Jousilahti P, Jula A, Kaakinen

M, Kajantie E, Kaplan LM, Kathiresan S, Kettunen J, Kinnunen L, Knowles JW, Kolcic I, Konig IR, Koskinen S, Kovacs P, Kuusisto J, Kraft P, Kvaloy K, Laitinen J, Lantieri O, Lanzani C, Launer LJ, Lecoeur C, Lehtimaki T, Lettre G, Liu J, Lokki M-L, Lorentzon M, Luben RN, Ludwig B, Manunta P, Marek D, Marre M, Martin NG, McArdle WL, McCarthy A, McKnight B, Meitinger T, Melander O, Meyre D, Midthjell K, Montgomery GW, Morken MA, Morris AP, Mulic R, Ngwa JS, Nelis M, Neville MJ, Nyholt DR, O'Donnell CJ, O'Rahilly S, Ong KK, Oostra B, Pare G, Parker AN, Perola M, Pichler I, Pietilainen KH, Platou CGP, Polasek O, Pouta A, Rafelt S, Raitakari O, Rayner NW, Ridderstrale M, Rief W, Ruokonen A, Robertson NR, Rzehak P, Salomaa V, Sanders AR, Sandhu MS, Sanna S, Saramies J, Savolainen MJ, Scherag S, Schipf S, Schreiber S, Schunkert H, Silander K, Sinisalo J, Siscovick DS, Smit JH, Soranzo N, Sovio U, Stephens J, Surakka I, Swift AJ, Tammesoo M-L, Tardif J-C, Teder-Laving M, Teslovich TM, Thompson JR, Thomson B, Tonjes A, Tuomi T, van Meurs JBJ, van Ommen G-J, Vatin V, Viikari J, Visvikis-Siest S, Vitart V, Vogel CIG, Voight BF, Waite LL, Wallaschofski H, Walters GB, Widen E, Wiegand S, Wild SH, Willemsen G, Witte DR, Witteman JC, Xu J, Zhang Q, Zgaga L, Ziegler A, Zitting P, Beilby JP, Farooqi IS, Hebebrand J, Huikuri HV, James AL, Kahonen M, Levinson DF, Macciardi F, Nieminen MS, Ohlsson C, Palmer LJ, Ridker PM, Stumvoll M, Beckmann JS, Boeing H, Boerwinkle E, Boomsma DI, Caulfield MJ, Chanock SJ, Collins FS, Cupples LA, Smith GD, Erdmann J, Froguel P, Gronberg H, Gyllensten U, Hall P, Hansen T, Harris TB, Hattersley AT, Hayes RB, Heinrich J, Hu FB, Hveem K, Illig T, Jarvelin M-R, Kaprio J, Karpe F, Khaw K-T, Kiemeney LA, Krude H, Laakso M, Lawlor DA, Metspalu A, Munroe PB, Ouwehand WH, Pedersen O, Penninx BW, Peters A, Pramstaller PP, Quertermous T, Reinehr T, Rissanen A, Rudan I, Samani NJ, Schwarz PEH, Shuldiner AR, Spector TD, Tuomilehto J, Uda M, Uitterlinden A, Valle TT, Wabitsch M, Waeber G, Wareham NJ, Watkins H, Wilson JF, Wright AF, Zillikens MC, Chatterjee N, McCarroll SA, Purcell S, Schadt EE, Visscher PM, Assimes TL, Borecki IB, Deloukas P, Fox CS, Groop LC, Haritunians T, Hunter DJ, Kaplan RC, Mohlke KL, O'Connell JR, Peltonen L, Schlessinger D, Strachan DP, van Duijn CM, Wichmann HE, Frayling TM, Thorsteinsdottir U, Abecasis GR, Barroso I, Boehnke M, Stefansson K, North KE, I McCarthy M, Hirschhorn JN, Ingelsson E and Loos RJF. Association analyses of 249,796 individuals reveal 18 new loci associated with body mass index. *Nat Genet*. 2010;42:937-948.

10. Stergiakouli E, Gaillard R, Tavaré JM, Balthasar N, Loos RJ, Taal HR, Evans DM, Rivadeneira F, St Pourcain B, Uitterlinden AG, Kemp JP, Hofman A, Ring SM, Cole TJ, Jaddoe VWV, Davey Smith G and Timpson NJ. Genome-wide association study of height-adjusted BMI in childhood identifies functional variant in ADCY3. *Obesity (Silver Spring, Md)*. 2014;22:2252-2259.
11. Stergiakouli E, Martin J, Hamshere ML, Heron J, St Pourcain B, Timpson NJ, Thapar A, Davey Smith G. Association between polygenic risk scores for attention-deficit hyperactivity disorder and educational and cognitive outcomes in the general population. *Int J Epidemiol* 2017;46:421-428
12. McCarron P, Smith GD, Okasha M, McEwen J. Blood pressure in young adulthood and mortality from cardiovascular disease. *The Lancet*;355(9213):1430-31. doi: 10.1016/S0140-6736(00)02146-2
